# Novel class of photochromic molecules exhibiting photo-switching in the solid state

**DOI:** 10.3389/fchem.2023.1205452

**Published:** 2023-06-07

**Authors:** Thomas Loan, Mithun Santra, Mark Bradley

**Affiliations:** School of Chemistry, University of Edinburgh, Edinburgh, United Kingdom

**Keywords:** photo-switching, chromogenic, cycloaddition, solid state, phenylindole alkene dimers

## Abstract

Photo-switching compounds are widely used as super-resolution imaging agents, anti-counterfeiting dyes, and molecules that are able to control drug–receptor interactions. However, advancement of this field has been limited by the number of classes of molecules that exhibit this phenomenon, and thus there are growing activities to discover new photo-switching compounds that diversify and improve current applications and include the so-called donor–acceptor Stenhouse adducts. Herein, a new class of compounds, phenylindole alkene dimers, are presented as a novel class of photochromic molecules that exhibit photo-switching in the solid state. The synthesis of a small library of these compounds allowed the tuning of their optical properties. Surfaces coated with these photo-switches can be used as writable materials in a variety of applications.

## Graphical Abstract

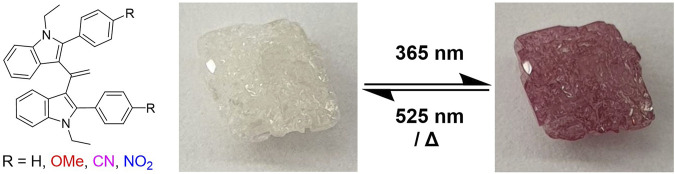

## 1 Introduction

Photo-switching molecules exhibit a reversible change in their absorbance spectrum upon irradiation, typically mediated by a photochemical molecular transformation, often in the form of photo-isomerisations, proton transfers, or pericyclic reactions. Molecules undergoing this type of transformation include azobenzenes (Merino and Ribagorda, 2012) and spiropyrans (Scarmagnani et al, 2008) typically changing from a colourless to a coloured state upon UV irradiation. For these molecules, the colour eventually disappears as the molecule switches back, as the switched state is thermodynamically unstable (so-called T-type photochromophores) (Irie et al, 2014). Other molecules such as furylfulgides (Yokoyama, 2000) and diarylethenes (Tian and Yang, 2004) are thermally stable once illuminated but may be converted back to the original colourless state upon irradiation with a different wavelength of light (so-called P-type photochromophores) (Irie et al, 2014). Other reported classes of photo-switches include donor–acceptor Stenhouse adducts (Helmy et al, 2014), napthopyrans (Sousa et al, 2012), and stilbenes (Waldeck, 1991).

Photo-switching compounds are widely used as super-resolution imaging agents (Dempsey et al, 2011) and molecules that can control drug–receptor interactions upon illumination (Mulatihan et al, 2020). Photochromic organic compounds that switch effectively either in solid matrixes or in the crystal state are quite limited (Irie, 2021). They have recently attracted attention for applications in data storage (Gonzalez et al., 2020) or as anti-counterfeiting dyes (Ju et al., 2020), and include compounds such as diarylethenes (Kobatake et al, 2004) and aziridines (DoMinh et al, 1979).

Herein, phenylindole alkene dimers (PIDs), a new class of photochromic molecules, are reported that exhibit rapid conversion from a colourless state to a coloured state, with both photo-reversibility and thermal reversibility in the solid state. The optical properties of these photochromes were studied in the solid state and applied as photo-switchable inks.

## 2 Results

### 2.1 Synthesis

**FIGURE 1:**
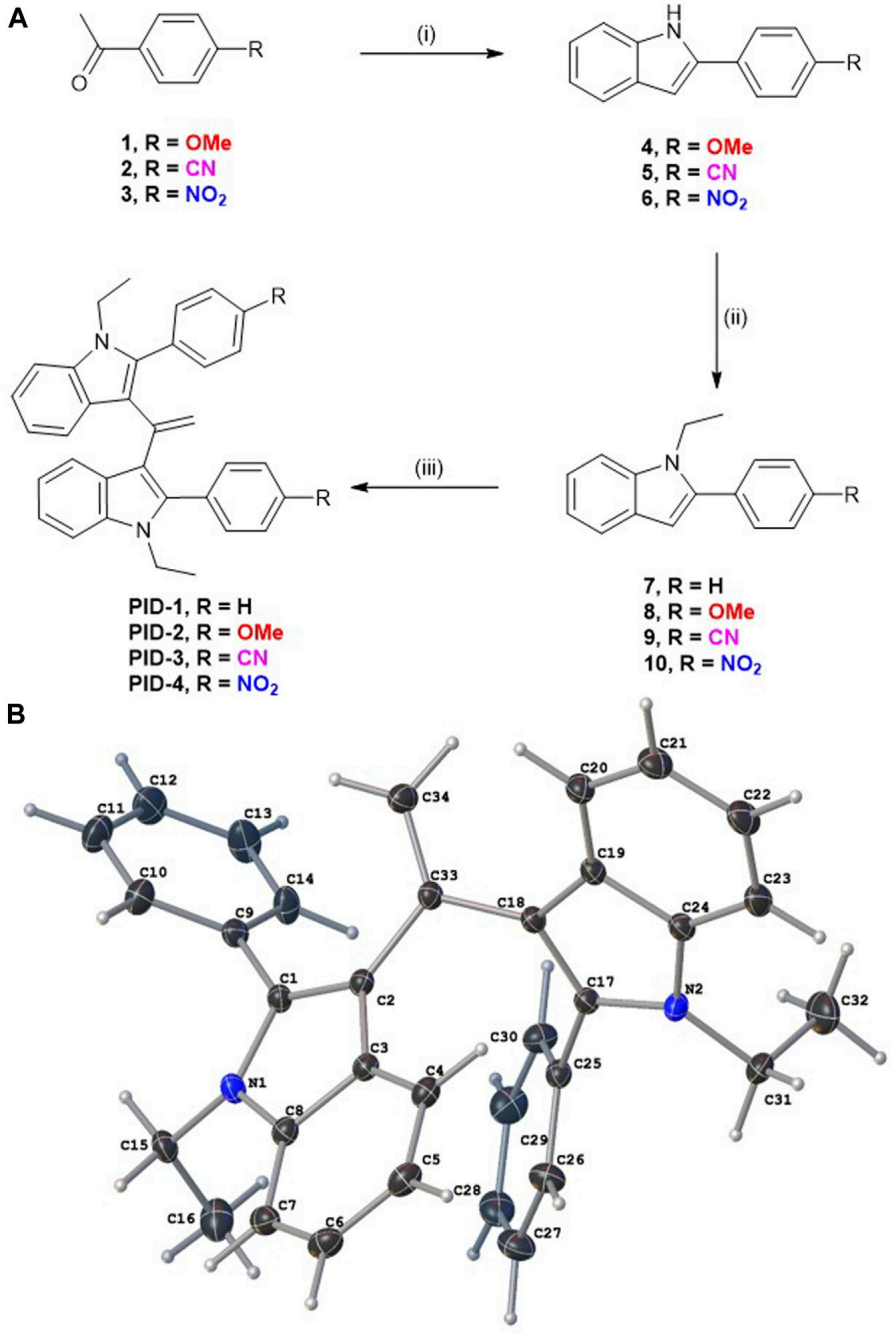
**(A)** Synthesis route for compounds **PID 1–4**. Reagents and conditions: **(I)** Phenylhydrazine (1.2 equiv.), CH_3_CO_2_H, 120 °C (microwave heating), 70–180 min, 64%–72%; (ii) EtBr (2 equiv.), Cs_2_CO_3_ (2 equiv.), acetonitrile, 75 °C, 13–16 h, 50%–96%; and (iii) CH_3_COCl (0.5–10 equiv.), CH_3_CO_2_H, 100 °C 2–19 h, 2%–64%. **(B)** Molecular structure of **PID-1**, as determined by single-crystal X-ray diffraction with the double bond C34=C33 clearly visible.

PID-1, 2, 3, and 4 were synthesised as shown in Figure 1A. Fischer indole synthesis afforded phenylindoles **4–6**, followed by N-alkylation with bromoethane. Commercially available, **7**, plus intermediate products **8–10** were dimerised via acetyl chloride-promoted Knoevenagel condensation (Noureddin et al, 2020). Their structures were confirmed by NMR analysis (see SI for the ^1^H and ^13^C NMR data) with a singlet at approximately 5.16 ppm, highly characteristic of gem-disubstituted olefin resonances. The materials were robust solids, typically white or off-white in colour except for the nitro analogue which was yellow (as is typically found for many nitro aromatics) (Porter, 1955). Single-crystal X-ray diffraction of PID-1 confirmed the molecular structure (Figure 1B).

### 2.2 Photo-switching in the solid state

**FIGURE 2:**
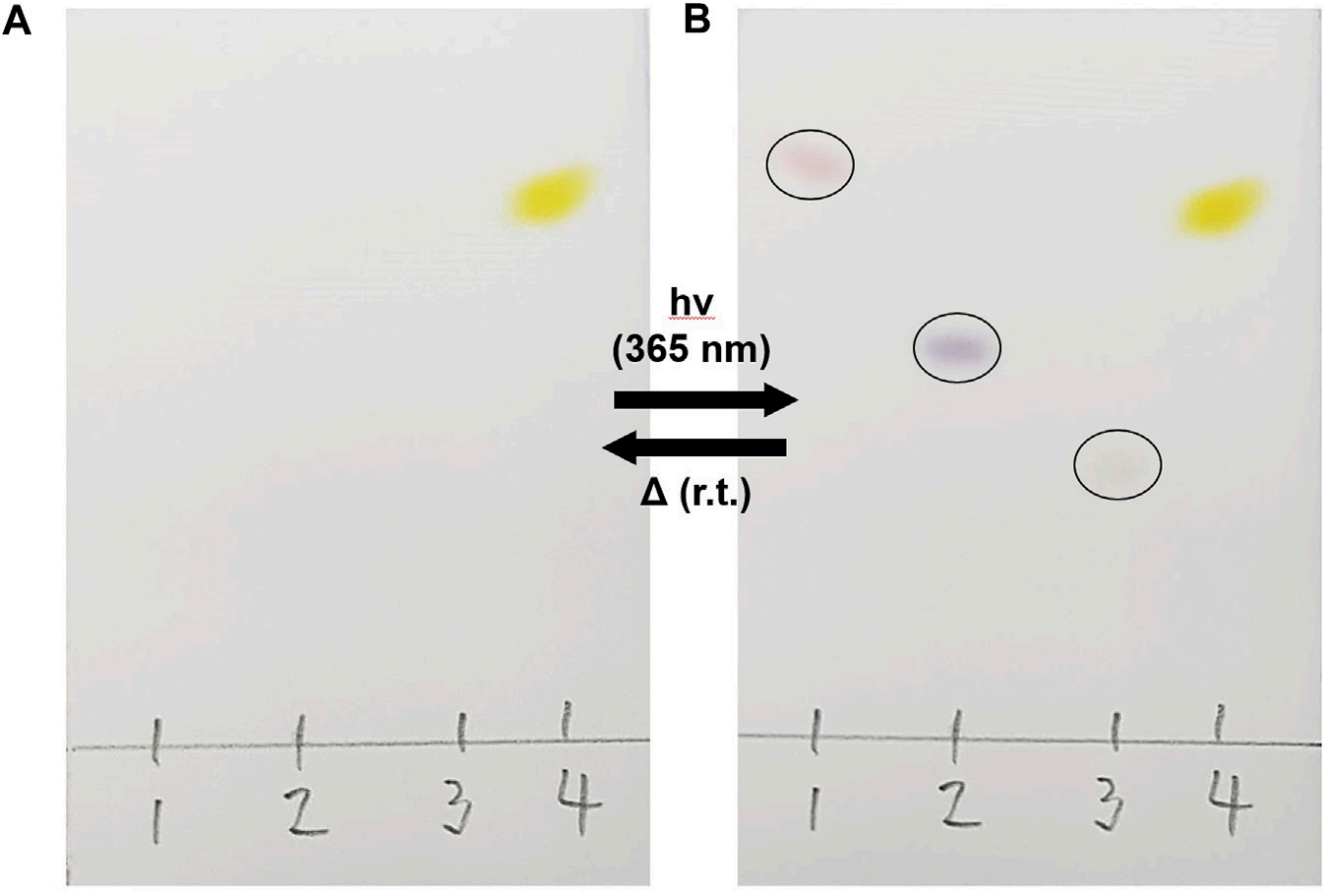
Light sensitivity of **PID 1–4**. Compounds were spotted onto silica gel TLC plates and eluted with 20% EtOAC/hexane. **(A)** Before and **(B)** after irradiation at 365 nm.

The switching behaviour of PID-1 was a serendipitous discovery, with its properties identified following the analysis of compound purity by thin-layer silica gel chromatography (TLC) and UV illumination (365 nm). Further analysis and synthesis showed that compounds **PID-1**, **PID-2**, and **PID-3** all exhibited light switching/sensitivity and interestingly showed a range of colours (pink, purple, and orange) upon switching (Figure 2). With the removal of the UV light, the colours would disappear under ambient conditions via thermal relaxation. However, the p-nitrophenyl derivative, **PID-4**, was yellow before and after irradiation, with no apparent photo-switching character at this wavelength.

### 2.3 Characterisation of the solid-state photo-reaction

#### 2.3.1 Optical characterisation

**FIGURE 3:**
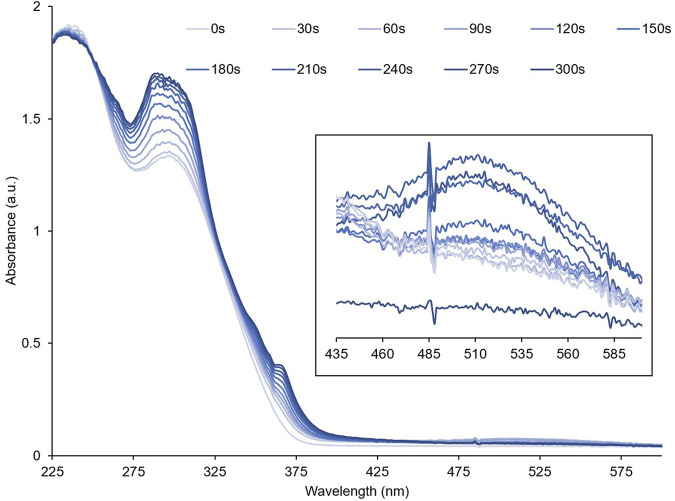
Absorbance spectra of a thin film of **PID-1** upon increasing irradiation at 365 nm. Inset shows the expansion from 435 to 600 nm.

**FIGURE 4:**
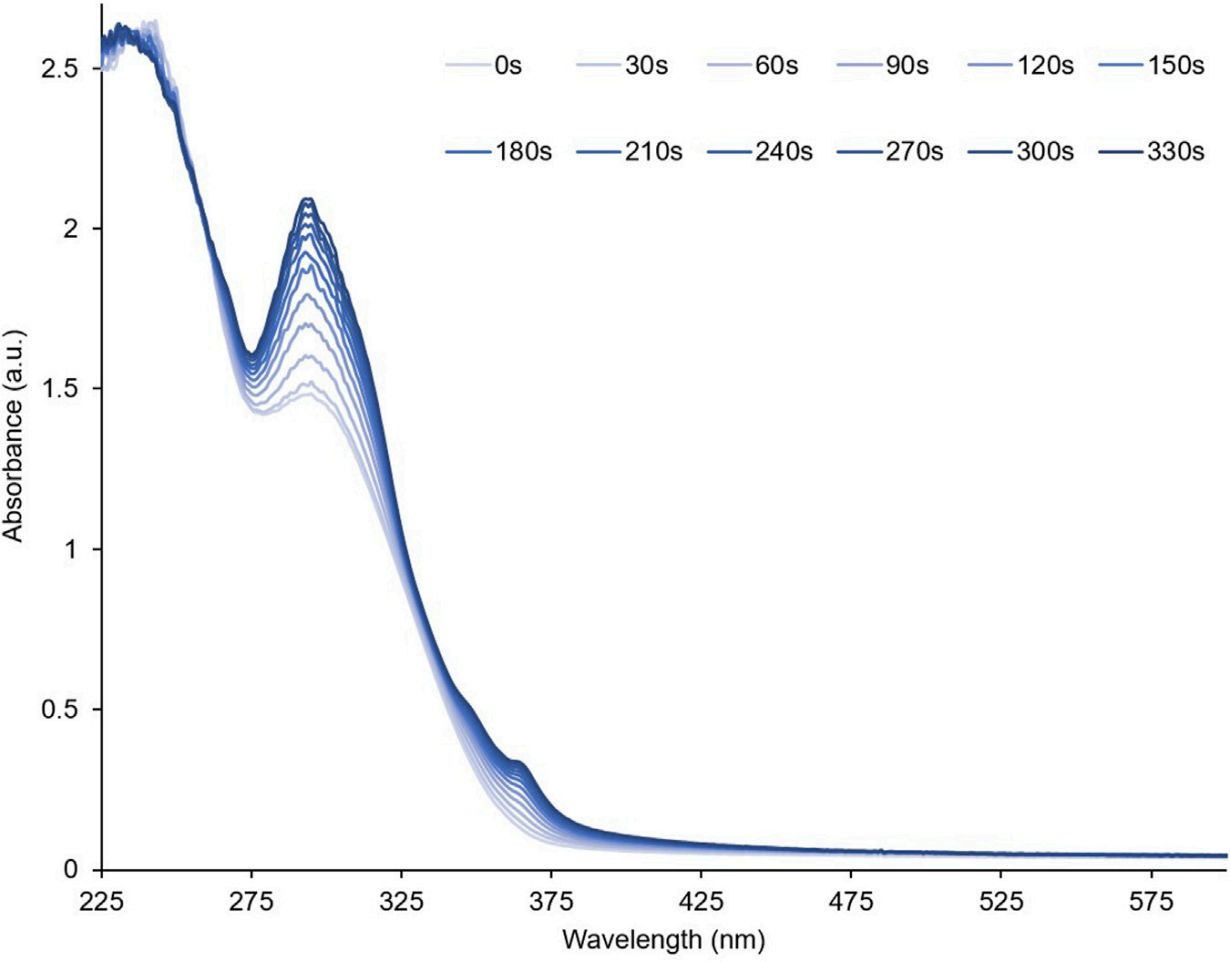
Absorbance spectrum of a thin film of **PID-2** upon increasing irradiation at 365 nm.

To investigate their optical properties, thin films of **PID-1**, **2**, and **3** were prepared by solvent coating onto quartz. **PID-1** showed strong UV absorption bands at 232 nm and 301 nm. Upon irradiation at 365 nm, there was an increase in the absorbance band at 301 nm and the generation of new broad band centred at 515 nm (Figure 3). This new band reached a maximum absorbance after 60 s of irradiation, but then gradually decreased upon further irradiation. This was in contrast to the band at 301 nm, which increased with the irradiation time reaching a plateau in intensity. With increasing irradiation, **PID-2** also exhibited an increase at 301 nm (Figure 4), but with no discernable increase within the visible region, in contrast to the colour previously observed by the TLC experiment (see Figure 2). Upon increasing irradiation, **PID-3** showed a decrease in a band at 261 nm, with an increase in absorbance bands at 317 nm and 394 nm (Supplementary Figure S25).

To demonstrate the reversibility of the photo-reaction, absorbance spectra of thin films of **PID-1** were collected before and after irradiation (60 s at 365 nm), followed by 15 s of irradiation at 525 nm (Supplementary Figure S23). Upon irradiation at 525 nm, the longer wavelength, disappeared, whilst the 301 nm band did not. Similar to photo-reversibility, the films exhibited slow thermal reversibility, with the 515 nm band decreasing over 15 min when in the dark (Supplementary Figure S24), reflecting the visual reversibility previously observed (see Figure 2).

**FIGURE 5:**
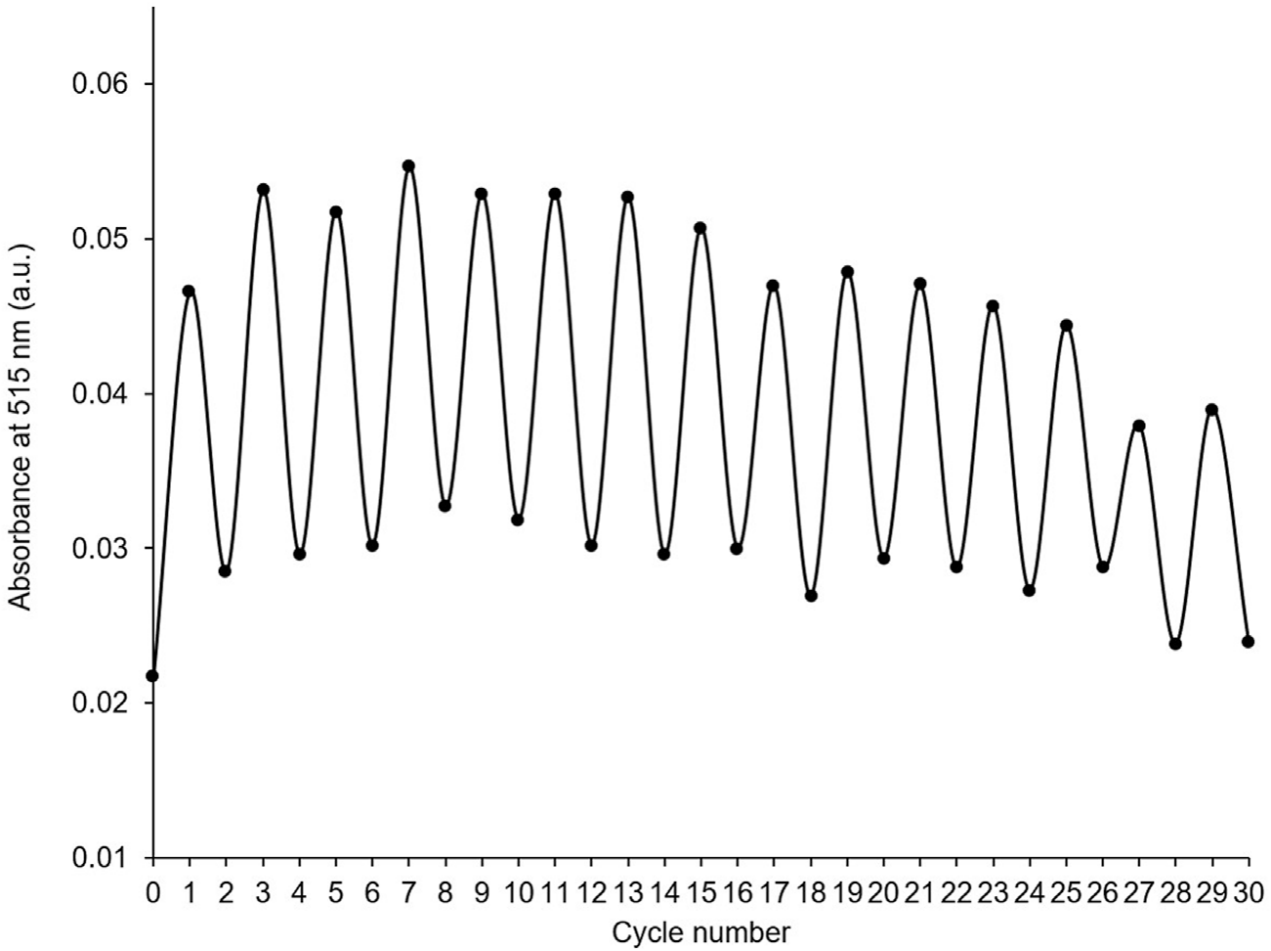
Absorbance at 515 nm of thin film of PID-1 after alternating cycles of irradiation with UV light (365 nm, 5 s), followed by UV-visible light (525 nm, 2s).

The stability of PID-1 over several switching cycles was tested by collecting absorbance spectra after alternate irradiation of a thin film of **PID-1** with UV light (365 nm, 5 s) and UV-visible light (525 nm, 2 s). The compound showed large differences in absorbance between switched states over 30 cycles with only a relatively minor decrease in intensity by the 30th cycle (Figure 5).

**FIGURE 6:**
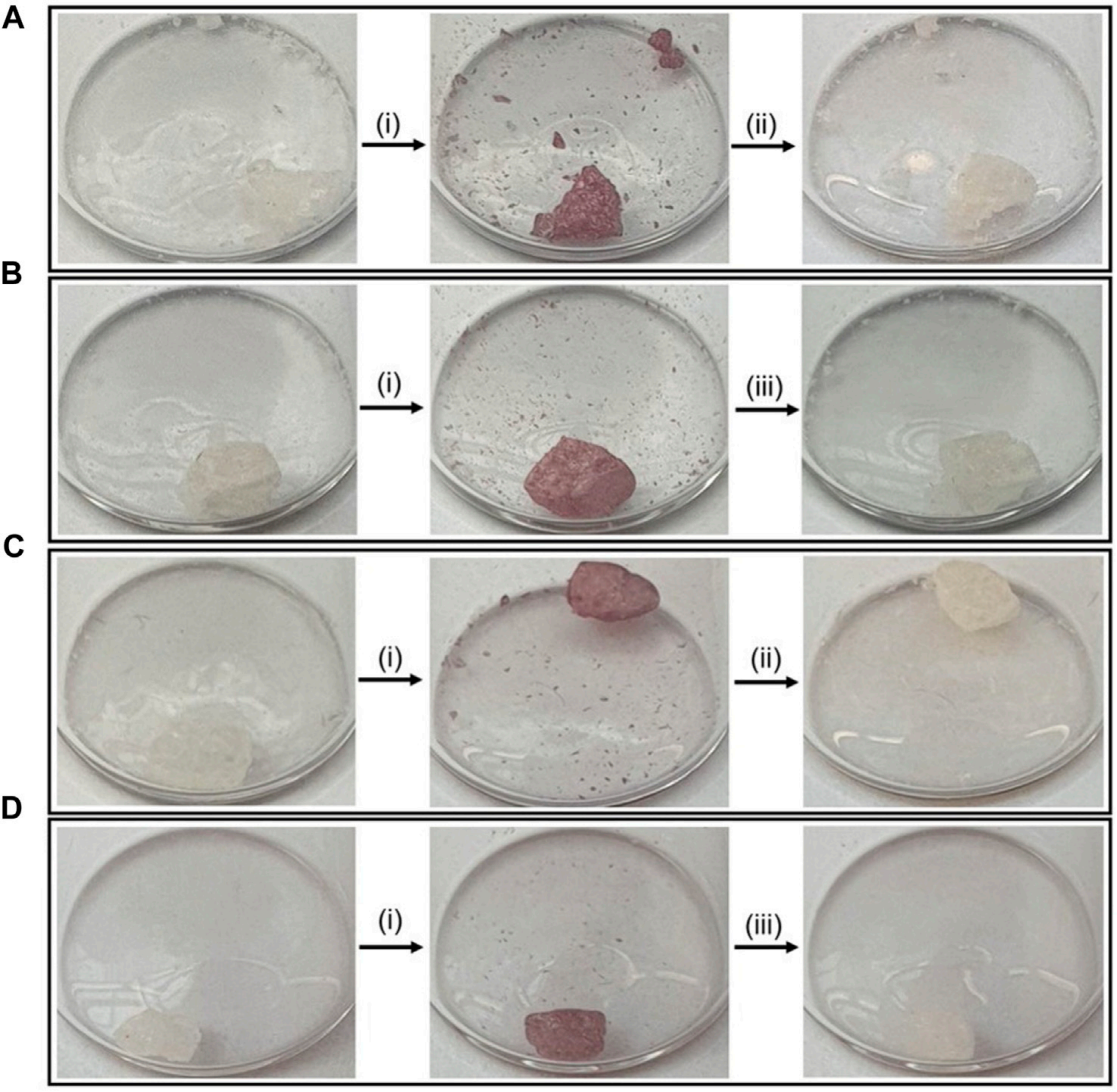
Photo-switching of **PID-1** under air or nitrogen. **(A)** Thermal reversibility under air, **(B)** photo-reversibility under air, **(C)** thermal reversibility under nitrogen, and **(D)** photo-reversibility under nitrogen. **(i)** Irradiation at 365 nm for 60 s, **(ii)** left in the dark for 17.5 h, and **(iii)** irradiation at 525 nm for 15 s.

To investigate whether the presence of oxygen had any effect on photo-switching, crystals of **PID-1** were illuminated in air as well as under N_2_ (Figure 6).

No difference was observed when under N_2_ in both switching to the coloured state and showing thermal reversibility and photo-reversibility, demonstrating that the mechanism of the photo-reaction was not oxygen-dependent.

#### 2.3.2 Structural characterisation

**FIGURE 7:**
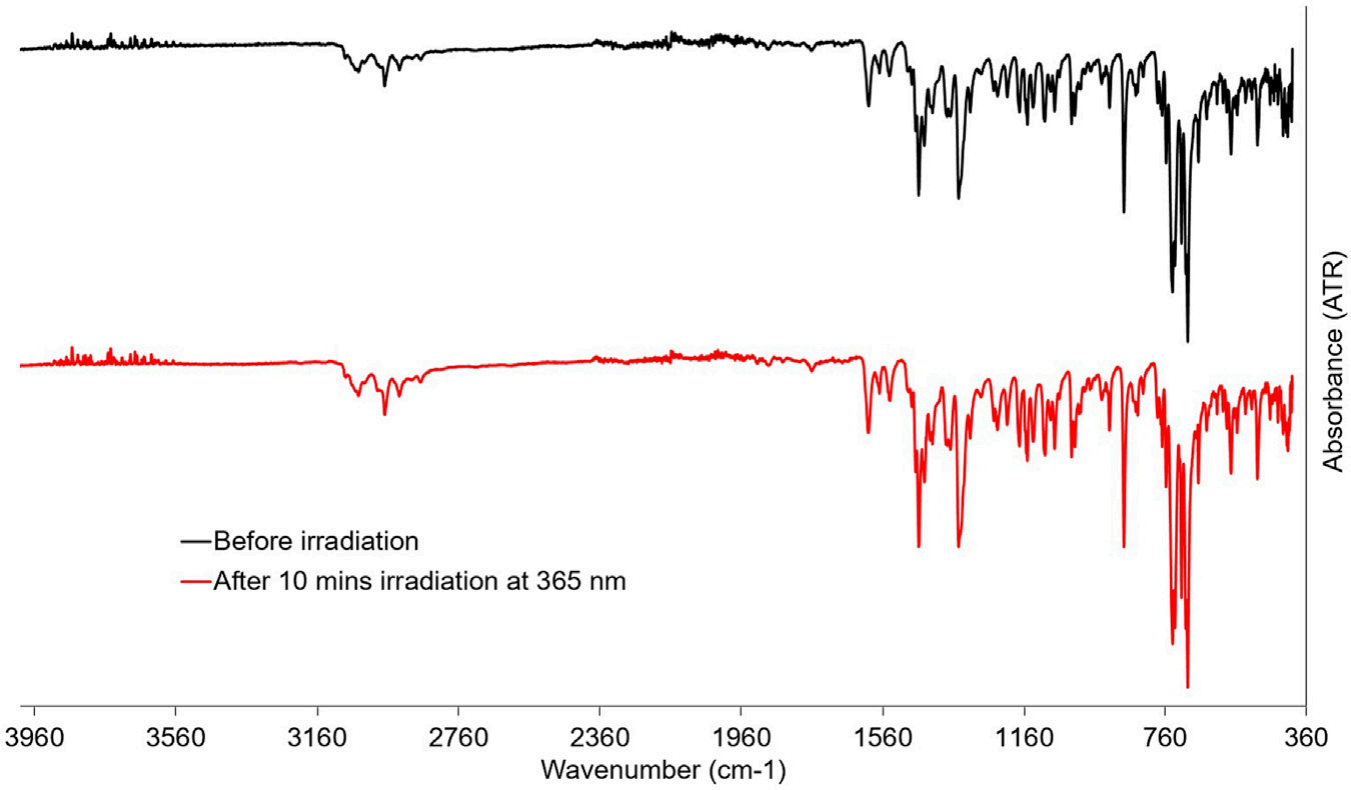
ATR-FTIR spectra of powder samples of PID-1 before and after irradiation at 365 nm.

In order to elucidate the structural changes upon irradiation, ATR-FTIR spectra of powder samples of PID-1 were taken before and after irradiation at 365 nm. However, no discernable changes in the spectra could be observed despite the observed colour change of the powder (Figure 7).

### 2.4 Photo-switchable inks for light-based pattern printing

**FIGURE 8:**
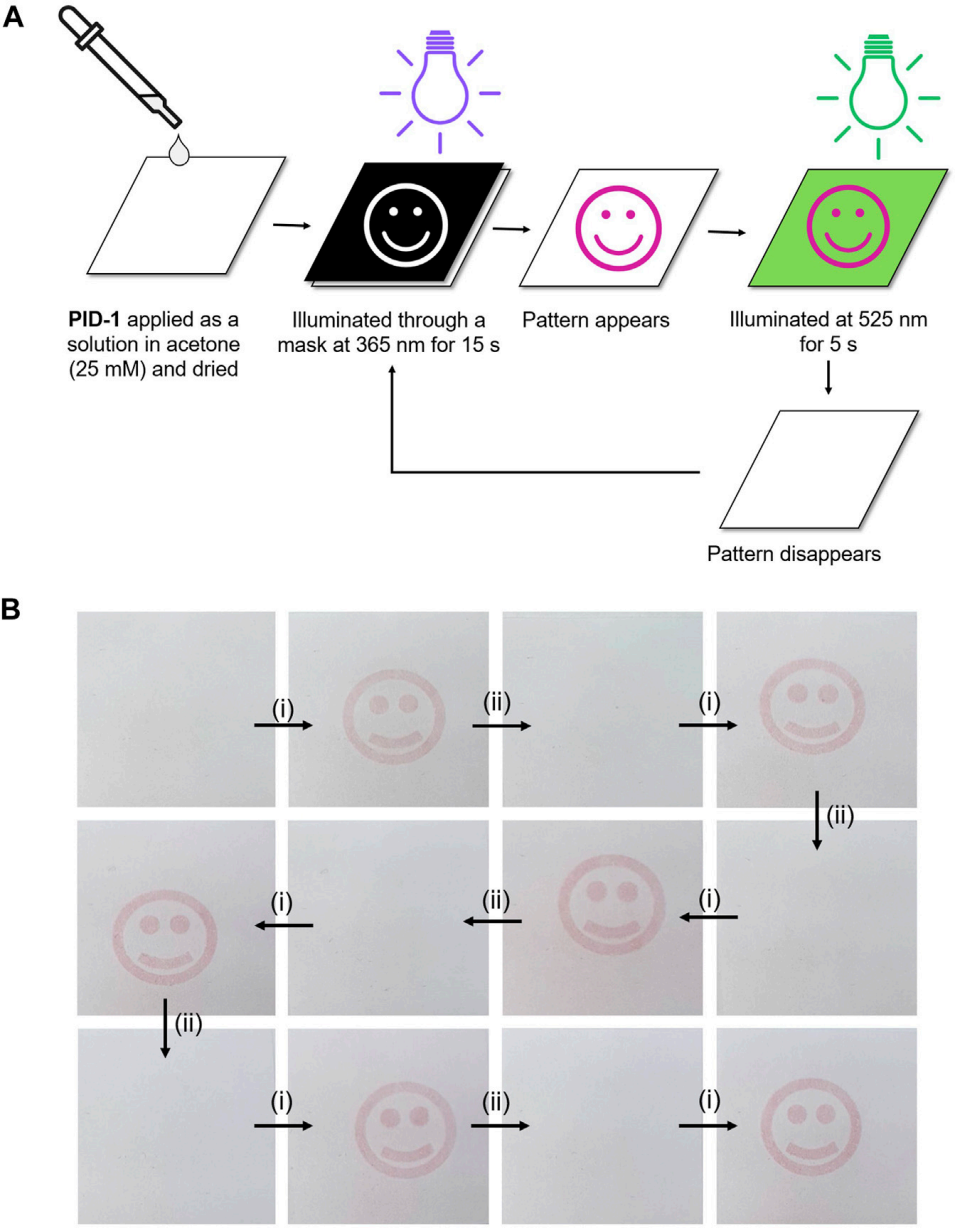
**(A)** Patterning using 365 nm illumination of **PID-1** through a mask. **(B)** Cycles of the generation and disappearance of images upon orthogonal illumination: **(i)** 365 nm illumination (15 s) and **(ii)** 525 nm illumination (5 s).

The application of **PID-1** as a light-responsive ink was demonstrated by patterned illumination of **PID-1** coated onto white printer paper. This was achieved by printing a black inverse mask onto an acetate transparency by inkjet printing which allowed light-induced patterning on the paper, with “pink/purple” images appearing, that remained visible for 15 min (Supplementary Figure S22). However, these patterns rapidly disappeared when irradiated at 525 nm. This process was fully reversible over many cycles without loss of colour intensity or photobleaching (Figure 8) (for video of photo-switching with photo and thermal reversibility, see Supplementary Video S1).

### 2.5 Discussion

**SCHEME 1:**
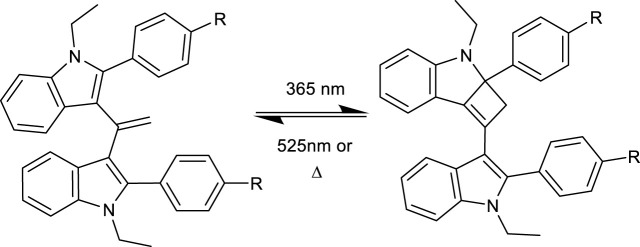
Proposed mechanism for the photo-reversibility and thermal reversibility reactions.

The pink/purple colour changes observed (see Figure 2) and absorbance measurements (showing a new band at approximately 515 nm) upon irradiation suggest that the product had an increased degree of conjugation. The fact that the reaction was light-induced suggested that the forward reaction could be a [2 + 2] cycloaddition, generating a strained cyclobutene unit upon reaction between the olefin and the indole (Scheme 1). This undergoes an allowed [2 + 2] reverse reaction when illuminated at 525 nm to regenerate the starting material. The room temperature (in the dark) reversion is slow, as a classical [2 + 2] reaction is “disallowed” explaining the relatively slow change seen. For example, when the compounds were illuminated as shown in Figure 2 in the dark, they took >15 min to return to the colourless state.

The proposed mechanism is in concurrence with evidence of other reported solid-phase photo-switches, such as diarylethenes (Kobatake et al, 1999) and nobornadienes (Bauer et al, 2019), with the transformation resulting in a relatively small change in volume of the molecule. It has been previously reported that small changes in volume between switched states is more favourable to solid-phase photo-switching due to the decreased steric hindrance of nearby molecules in the crystals (Gonzalez et al, 2020). We hypothesize that a reversible [2 + 2] cycloaddition is responsible for the photo-reversible reaction, but further studies are required to fully understand this reaction, perhaps by *in situ* illumination solid-state NMR spectroscopy. Other mechanistic options for the thermally reversible route include via a bis-radical or an indole cation/allylic anion.

Of the synthesised compounds, **PID-1** showed the most promising properties due to its one-step synthesis, its stability in aerobic environments, and the large difference between the switched and unswitched states in the visible region of the absorption spectra. Its application as a photochromic ink was demonstrated with many cycles of writing (365 nm illumination) and erasing (535 nm illumination).

Possible future applications of these materials include writable filters or gratings. Within a biological context, **PIDs** could find applications in modulating drug–receptor interactions.

## 3 Methods and materials

### 3.1 General information

Irradiation of samples at 365 nm was performed using an ENF-240C E-Series UV Lamp (4 W, 120 V). Irradiation of samples at 525 nm was performed using a Thorlabs LIU525B-525 nm Green LED Array Light Source.

### 3.2 Photo-switching on paper

A solution of **PID-1** (25 mM in acetone) was drop-coated onto A4 standard white printer paper (purchased from Banner). The solvent was evaporated under a nitrogen flow. Masks were prepared by inkjet printing patterns onto A4 acetate transparencies (purchased from Niceday) and cut to a size of 7.5 cm × 6 cm.

The paper and mask were sandwiched together and irradiated (365 nm, 15 s). The treated sample was either irradiated (525 nm, 5 s) or left in the dark (15 min).

### 3.3 Absorbance spectroscopy

Absorbance spectra were collected from 190 nm to 700 nm on an Agilent 8453 UV-visible absorbance spectrometer (integration time = 0.5 s). Thin films of the solid compound were prepared as follows: 50 µL of **PID** in DCM (38 mM) was placed onto one side of the 3-mL quartz cuvette (path length 1 cm) which was evaporated under N_2_. Spectra for **PID-1**, **2**, and **3** were collected with an increasing irradiation time (365 nm). Furthermore, spectra of **PID-1** were collected before and after irradiation (365 nm, 2 min) and then either left in the dark for 15 min or irradiated at 525 nm for 15 s.

Cycles of 365 nm and 525 nm on thin films: Thin films of PID-1 were prepared as previously described, however with addition of 2 × 50 µL (38 mM, DCM) of PID-1. Spectra were collected before irradiation and then after 30 cycles of UV irradiation (365 nm, 5 s) and UV-visible irradiation (525 nm, 2 s).

### 3.4 ATR-FTIR spectroscopy

ATR-FTIR spectroscopy was performed on a Perkin Elmer Spectrum Two (UAR Two) FTIR spectrometer. Spectra were taken on powder samples of **PID-1** at 300 K before and after irradiation at 365 nm for 10 min.

### 3.5 Irradiation of PID-1 samples under air/nitrogen

Samples of **PID-1** (4 mg) were added to glass vials (1.7 mL). Two samples were left in air, and two samples were purged and backfilled with N_2_ (×3 times). The samples were irradiated at 365 nm for 60 s and then either left in the dark for 17.5 h or irradiated at 525 nm for 15 s.

### 3.6 Single-crystal X-ray diffraction

**PID-1** was recrystallised from a mixture of ethyl acetate and hexane by slow evaporation. A suitable crystal with dimensions 0.42 × 0.30 × 0.27 mm^3^ was selected and mounted on a MITIGEN holder in paratone oil on a Rigaku Oxford Diffraction Xcalibur diffractometer. The crystal was kept steady at T = 120.01 (10) K during data collection. The structure was solved using the SHELXS solution program by using direct methods and Olex2-1.5-beta as the graphical interface. The model was refined with SHELXL 2018/3 using full matrix least squares minimisation on F2. The crystallography data were uploaded to the Crystallography Open Database, under accession number 3000437.

### 3.7 Synthesis of compounds 1–4

For full synthetic procedure and characterisation by NMR spectroscopy and mass spectrometry, see the Supplementary Material section.

## Data Availability

The datasets presented in this study can be found in online repositories. The names of the repository/repositories and accession number(s) can be found at: https://www.crystallography.net/cod/index.php, 3000437.
