# Supplementary material for: Novel class of photochromic molecules exhibiting photo-switching in the solid state
Source: Front Chem. 2023 Jun 7;11:1205452. doi: 10.3389/fchem.2023.1205452 (PMC10282750; doi:10.3389/fchem.2023.1205452)
Supplement: Supplementary file 3 [file DataSheet1.docx]

Supplementary Material

Novel class of photochromic molecules exhibit photo-switching in the solid-state

Thomas Loan, Mithun Santra, Mark Bradley*

*** Correspondence:** Prof. Mark Bradley, mark.bradley@ed.ac.uk

**1 Supplementary information**

General methods: Unless otherwise noted, commercial reagents were purchased from Fluorochem, Aldrich, Merck, Acros, and other commercial suppliers and were used as received. Synthetic reactions were monitored using TLC Merck 60 F254 precoated silica gel plates. Normal phase, flash column chromatography was conducted on a Biotage Isolera fitted with a 254 nm detector. Nuclear magnetic resonance spectra (^1^H) were recorded on Bruker 500 or 600 MHz spectrometers in D_2_-DCM or CDCl_3_ unless stated otherwise (^13^C NMR 125 or 150 MHz). The chemical shift for the ^1^H NMR spectra is reported as δ in parts per million (ppm). Multiplicities are given as s (singlet), d (doublet), t (triplet), q (quartet), dd (doublet of doublet), dt (doublet of triplet), and m (multiplet). Coupling constants J are reported in Hertz.

High Resolution MS were performed on a Bruker microTOF focus II mass spectrometer.

Analytical reverse-phase high-performance liquid chromatography-evaporative light-scattering detector (HPLC-ELSD) was performed on an Agilent 1100 system equipped with a Phenomenex Kinetex® 5 µm XB-C18 100 Å LC Column (50 × 4.6 mm) with a flow rate of 1 mL/min, and a gradient of H_2_O/CH_3_CN (95/5) to H_2_O/CH_3_CN (20/80) with 0.1% CF_3_COOH, over 10 min, then to H_2_O/CH_3_CN (5/95), over 4 min, followed by 1 min isocratic elution with detection at 254 nm and by evaporative light scattering (ELSD).

**1.1 Synthetic Procedure**

**Synthesis of PID-1**

SI Fig. 1: Structure of **PID-1**

1-ethyl-2-phenylindole (2.0 g, 9.04 mmol) and acetic acid (12.5 mL) were added to a sealable glass vial. Acetyl chloride (1.92 mL, 18.1 mmol) was added and the vial was sealed with a crimp-able cap, purged and backfilled with N_2_. The flask was heated at 80 °C for 2 h, then poured slowly into iced water. The mixture was neutralized with sat. NaHCO_3_ and extracted with EtOAc (3 x 10 mL). The organic fractions were collected, dried over Mg_2_SO_4_, and filtered. The solvent was removed under vacuum, and the crude product was purified by flash chromatography (eluent = 10% EtOAc / Hexane) to yield the product as white crystals (0.94 g, 22%) ^1^H NMR (400 MHz, CDCl_3_) δ 7.47 (d, J = 6.9 Hz, 2H), 7.30 (d, J = 8.2 Hz, 2H), 7.18 (m 8H), 7.05 (m, 6H), 5.23 (s, 2H), 3.93 (q, J = 7.1 Hz, 4H), 1.12 (t, J = 8.0 Hz, 6H). ^13^C NMR (400 MHz, CDCl_3_) δ 137.2, 135.7, 134.8, 132.5, 130.4, 128.1, 127.4, 121.3, 120.5, 119.5, 116.7, 116.4, 109.2, 38.2, 15.2. HRMS (ESI)+ calcd for C_34_H_30_N_2_ [M + H]+ m/z 467.24818, found 467.2469. HPLC-ELSD t_R_ = 8.18 mins.

**Synthesis of 4**

SI Fig. 2: Structure of 4

A microwave vial was charged with 4-methoxyacetophenone (2.0 g, 13.3 mmol) and acetic acid (5 mL). Phenylhydrazine (1.57 mL, 16.0 mmol) was added along with a stir bar and the vial was sealed with a crimp-able cap. The mixture was heated in a Biotage microwave reactor (120 °C, 70 mins). The solvent was removed under vacuum to yield the crude product as a light-yellow solid. The solid was recrystallized from hot ethanol, then washed with cold ethanol to yield the product as an off-white solid (2.13 g, 72%). ^1^H NMR (400 MHz, CDCl_3_) δ 7.75 (d, J = 8.9 Hz, 2H), 7.28 (t, J = 8.0 Hz, 2H), 7.11 (d, J = 8.7 Hz, 2H), 6.93 (d, J = 8.9 Hz, 2H), 6.8 (t, J = 7.2 Hz, 1H). 3.88 (s, 3H). ^13^C NMR (400 MHz, CDCl_3_) δ 159.6, 145.5, 131.9, 130.5, 129.2, 126.8, 119.8, 113.7, 113.1, 58.3

**Synthesis of 8**

SI Fig. 3: Structure of 8

A sealable vial was charged with a solution of 4 (0.50 g, 2.24 mmol) in anhydrous acetonitrile (10 mL). Bromoethane (322 µL, 4.48 mmol) was added along with Cs_2_CO_3_ (1.46 g, 4.48 mmol). The vial was sealed with a crimp-able cap and heated in an oil bath at 60 °C for 16h. The mixture was cooled to room temperature, diluted with H_2_O (10 mL), then extracted with EtOAc (3 x 20 mL). The collected organic fractions were dried over Mg_2_SO_4_, filtered, and the solvent removed under vacuum to yield an orange / brown oil. The crude product was purified by flash chromatography (eluent = 10% EtOAc / Hexane) to yield the product as a yellow solid (0.283 g, 50%). ^1^H NMR (400 MHz, CDCl_3_) δ 7.92 (d, J = 8 Hz, 2H) 7.28 (t, J = 8 Hz, 2H), 6.98 (d, J = 8 Hz, 2H), 6.94 (d, J = 8 Hz, 1H), 6.91 (t, J = 8 Hz, 1H), 6.34, (q, J = 8 Hz, 2H), 2.23 (s, 3H), 1.26 (t, J = 8 Hz, 3H). ^13^C NMR (400 MHz, CDCl_3_) δ 166.0, 161.1, 150.4, 131.2, 128.9, 128.2, 120.2, 117.2, 113.7, 60.4, 55.39, 51.8, 16.4,

**Synthesis of PID-2**

SI Fig. 4: Structure of **PID-2**

To a sealable vial was added 8 (0.32 g, 1.22 mmol) along with acetic acid (3 mL). Acetyl chloride (190 µl, 2.66 mmol) was added, and the vial was sealed with a crimp-able cap, purged and backfilled with N_2_. The mixture was heated in an oil bath at 90 °C for 21 h. The mixture was cooled to room temperature, then slowly poured into ice water. The mixture was neutralized with sat. NaHCO_3_ and extracted with EtOAc (3 x 10 mL). The organic fractions were collected, dried over Mg_2_SO_4_, and filtered. The solvent was removed under vacuum, and the crude product was purified by flash chromatography (eluent = 10% EtOAc / Hexane) to yield the product as an off-white solid (0.117 g, 18%). ^1^H NMR (500 MHz, CDCl_3_) δ 7.52 (d, J = 8.0 Hz, 2H), 7.33 (d, J= 8.2 Hz, 2H), 7.22 (t, J = 8.2 Hz, 2H), 7.08 (t, J = 8.0 Hz, 2H), 7.02 (d, J = 8.8 Hz, 4H), 6.72 (d, J = 8.8 Hz, 4H), 5.32 (s, 2H), 3.97 (q, J = 7.2 Hz, 4H), 3.76 (s, 6H), 1.16 (t, J= 7.2 Hz, 6H). ^13^C NMR (500 MHz, CDCl_3_) δ 159.0, 137.1, 135.7, 135.2, 131.6, 128.2, 124.7, 121.2, 120.5, 119.4, 116.6, 116.1, 113.0, 109.2. HRMS (ESI)+ calcd for C_36_H_34_N_2_O_2_ [M + H]^+^ m/z 527.26930, found 527.2699. HPLC-ELSD t_R_ = 7.91 mins.

**Synthesis of 5**

SI Fig. 5: Structure of 5

A sealable microwave vial was charged with 4-cyanophenyl methyl ketone (2.0 g, 15.3 mmol) and acetic acid (6 mL). Phenylhydrazine (1.625 mL, 16.53 mmol) was added along with a stir bar and the vial was sealed with a crimp-able cap. The mixture was heated in a Biotage microwave reactor (125 °C, 180 mins). The mixture was cooled to room temperature, and was neutralized with saturated NaHCO_3_. The yellow precipitate was collected by filtration and washed with H_2_O, then dissolved in EtOAc (20 mL), dried over Mg_2_SO_4_, filtered, and the solvent removed under vacuum. The resulting crude product was recrystallized from hot ethanol, then washed with cold ethanol to yield the product as a yellow solid (2.34 g, 70%). ^1^H NMR (600 MHz, CDCl_3_) δ 7.91 (d, J = 8.7 Hz, 2H), 7.67(d, J = 8.7 Hz, 2H), 7.57 (s, 1H), 7.34 (t, J = 7.3 Hz, 2H), 7.22 (d, J = 8.7 Hz, 2H), 6.96 (t, J= 7.3 Hz, 1H). ^13^C NMR (400 MHz, CDCl_3_) δ 144.4, 143.3, 132.1, 129.4, 125.7, 121.0, 119.2, 113.4, 110.7

**Synthesis of 9**

SI Fig. 6: Structure of 9

A sealable vial was charged with a solution of 5 (1.0 g, 4.7 mmol) in anhydrous acetonitrile (10 mL). Bromoethane (680 µL, 9.17 mmol) was added along with Cs_2_CO_3_ (2.99 g, 9.18 mmol). The vial was sealed with a crimp-able cap and heated at 73 °C for 16 h. The mixture was cooled to room temperature, diluted with H_2_O (10 mL), then extracted with EtOAc (3 x 20 mL). The collected organic fractions were dried over Mg_2_SO_4_, filtered, and the solvent removed under vacuum to yield a red oil. The crude product was purified by flash chromatography (Eluent = 15% EtOAc / Hexane) to yield the product as a red solid (1.09 g, 96%). ^1^H NMR (400 MHz, CDCl_3_) δ 7.99 (d, J = 8.8 Hz, 2H), 7.71 (d, J = 8.7 Hz, 2H), 7.29 (t = 7.3 Hz, 2H), 6.99 (t, J = 7.3 Hz, 1H), 6.95 (d, J = 8.7 Hz, 2H), 3.66 (q, J = 7.0 Hz, 2H), 1.26 (t, J = 7.0 Hz). ^13^C NMR (400 MHz, CDCl_3_) δ 159.8, 150.3, 142.9, 132.1 129.1, 126.8, 122.0, 119.3, 118.9, 112.5, 60.4, 53.9, 17.0, 13.0

**Synthesis of PID-3**

SI Fig. 7: Structure of PID-3

To a sealable vial, 9 (0.465 g, 2.04 mmol) was added along with acetic acid (3.0 mL). Acetyl chloride (67 µl, 1.02 mmol) was added, and the flask was sealed with a crimp-able cap, purged and backfilled with N_2_. The mixture was heated at 90 °C for 18 h, cooled to room temperature, then slowly poured into ice water. The mixture was neutralized with sat. NaHCO_3_ and extracted with EtOAc (3 x 10 mL). The organic fractions were collected, dried over Mg_2_SO_4_, and filtered. The solvent was removed under vacuum, and the crude product was purified by flash chromatography (eluent = 15% EtOAc / Hexane) to yield the product as an off-white solid (5 mg, 2%). ^1^H NMR (500 MHz, CDCl_3_) δ 7.65 (d, J = 8.3 Hz, 3H), 7.49 (d, J = 7.1 Hz, 4H), 7.45 (d, J = 8.3 Hz, 3H), 7.40 (d, J = 8.4 Hz, 3H), 7.34 (t, 8.3 Hz, 3H), 7.15 (t, J = 7.1 Hz), 5.75 (d, J = 1.2 Hz, 1H), 5.36 (d, J = 1.2 Hz, 1H), 4.16 (q, J = 7.2 Hz, 4H), 1.33 (t, J = 7.2 Hz, 6H). ^13^C NMR (400 MHz, CDCl_3_) δ 146.0, 140.6, 136.7, 136.6, 132.1, 132.0, 131.0, 127.7, 127.5, 123.0, 120.6, 120.4, 119.7, 118.9, 118.4, 115.4, 112.0, 111.0, 110.2, 60.4, 39.1, 21.0, 15.4. HPLC-ELSD t_R_ = 6.75 mins

**Synthesis of 6**

SI Fig. 8: Structure of 6

A sealable microwave vial was charged 4-nitroacetophenone 2.5 g, 15.2 mmol) and acetic acid (5 mL). Phenylhydrazine (1.785 mL, 18.181 mmol) was added along with a stir bar and the vial was sealed with a crimp-able cap. The mixture was heated in a Biotage microwave reactor (125 °C, 180 mins). The mixture was cooled to room temperature, and was neutralized with saturated NaHCO_3_. The yellow precipitate was filtered and washed with H_2_O, then dissolved in EtOAc, and dried over Mg_2_SO_4_, filtered, and the solvent removed under vacuum. The resulting crude product was purified by flash chromatography (Eluent = 15% EtOAc / Hexane) to yield the product as a yellow solid (2.32 g, 64%). ^1^H NMR (400 MHz, CDCl_3_) δ 8.24 (d, J = 9.0 Hz, 2H), 7.96 (d, J = 9.0 Hz, 2H), 7.64 (s, 1H), 7.35 (t, J = 7.3 Hz, 2H), 7.23 (d, J = 8.8 Hz, 2H), 6.98 (t, J = 7.3 Hz). ^13^C NMR (400 MHz, CDCl_3_) δ 146.9, 145.1, 144.3, 137.9, 129.4, 125.8, 123.72, 121.2, 113.5.

**Synthesis of 10**

SI Fig. 9: Structure of 10

A sealable vial was charged with a solution of 6 (1.51 g, 6.39 mmol) in anhydrous acetonitrile (10 mL). Bromoethane (946 µL, 12.78 mmol) was added along with Cs_2_CO_3_ (4.166 g, 12.78 mmol). The flask was sealed with a crimp-able cap and heated at 75 °C for 13 h. The mixture was cooled to room temperature, diluted with H_2_O (10 mL), then extracted with EtOAc (3 x 20 mL). The collected organic fractions were dried over Mg_2_SO_4_, filtered, and the solvent removed under vacuum to yield a red oil. The crude product was purified by flash chromatography (eluent = 15% EtOAc / Hexane) to yield the product as a yellow oil (1.60 g, 93%).^1^H NMR (400 MHz, CDCl_3_) δ 8.26 (d, J = 9.0 Hz, 2H), 8.04 (d = 9.0 Hz, 2H), 7.31 (d, J = 7.4 Hz, 2H), 7.00 (t, J = 7.4 Hz, 1H), 6.97 (d, J = 9.0 Hz, 2H), 3.69 (q, J = 7.1 Hz, 2H), 1.28 (t, J = 7.1 Hz, 3H). ^13^C NMR (400 MHz, CDCl_3_) δ 158.7, 150.2, 148.1, 144.7, 129.1, 127.0, 123.6, 122.2, 119.6, 54.1, 13.1

**Synthesis of PID-4**

SI Fig. 10: Structure of **PID-4**

To a sealable flask was added 4b (111 mg, 0.417 mmol) along with acetic acid (1 mL). Acetyl chloride (297 µl, 4.17 mmol) was added, and the flask was sealed, purged and backfilled with N_2_. The mixture was heated at 87 °C for 3 h, cooled to room temperature, then slowly poured into ice water. The mixture was neutralized with sat. NaHCO_3_ and extracted with EtOAc (3 X 5 mL). The organic fractions were collected, dried over Mg_2_SO_4_, and filtered. The solvent was removed under vacuum, and the crude product was purified by flash chromatography (Eluent = 15% EtOAc / Hexane) to yield the product as yellow crystals (59 mg, 25%). ^1^H NMR (600 MHz, CDCl_3_) δ 8.36 (d, J = 8.8 Hz, 4H), 7.71 (d, J = 8.8, 3H), 7.70 (t, J = 7.1, 3H), 7.45, (d, J = 8.3, 2H), 7.32 (t, J = 8.3, 2H), 7.20 (t, J = 8.0 Hz, 2H), 6.69 (s, 2H), 4.27 (q, J = 7.2 Hz, 4H), 1.38 (t, J = 7.2 Hz, 6H). ^13^C NMR (600 MHz, CDCl_3_) δ 147.1, 139.7, 138.4, 138.0, 129.6, 128.1, 123.9, 122.8, 121.2, 120.4, 110.2, 104.5, 39.1, 15.4. HPLC-ELSD t_R_ = 6.51 mins.

**1.2 NMR/MS and HPLC data.**

**1.2.1 NMR spectra of PID- 1-4**


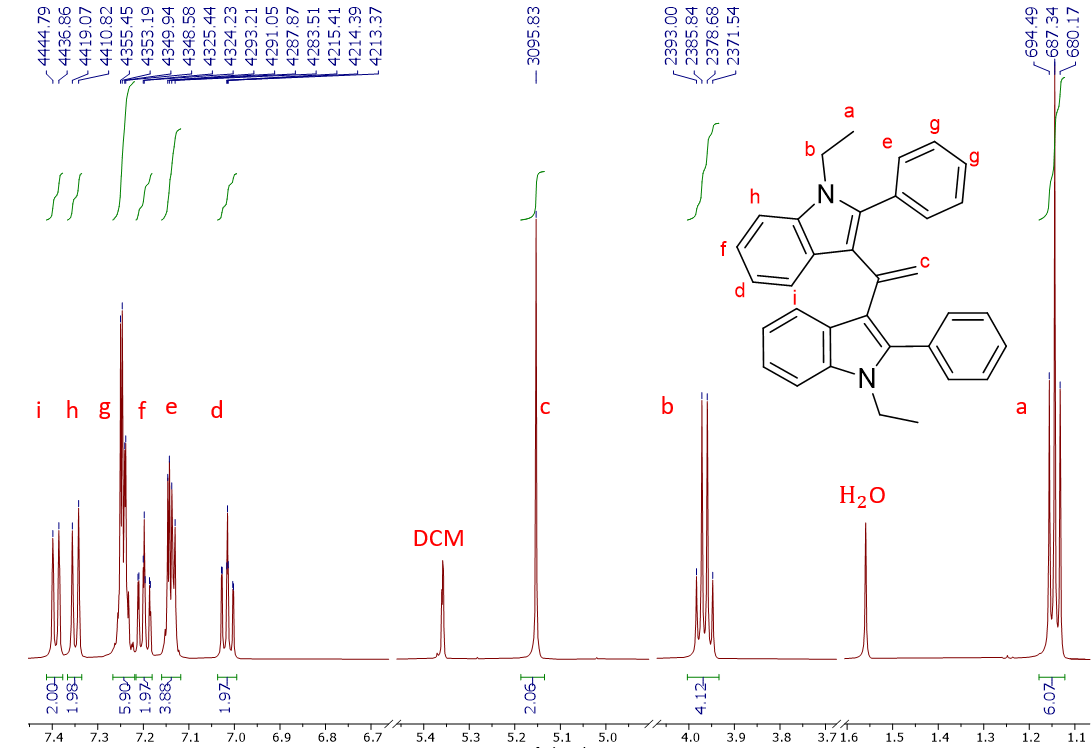


SI Fig. 11: ^1^H NMR characterisation of PID-1 in D_2_-DCM.

**
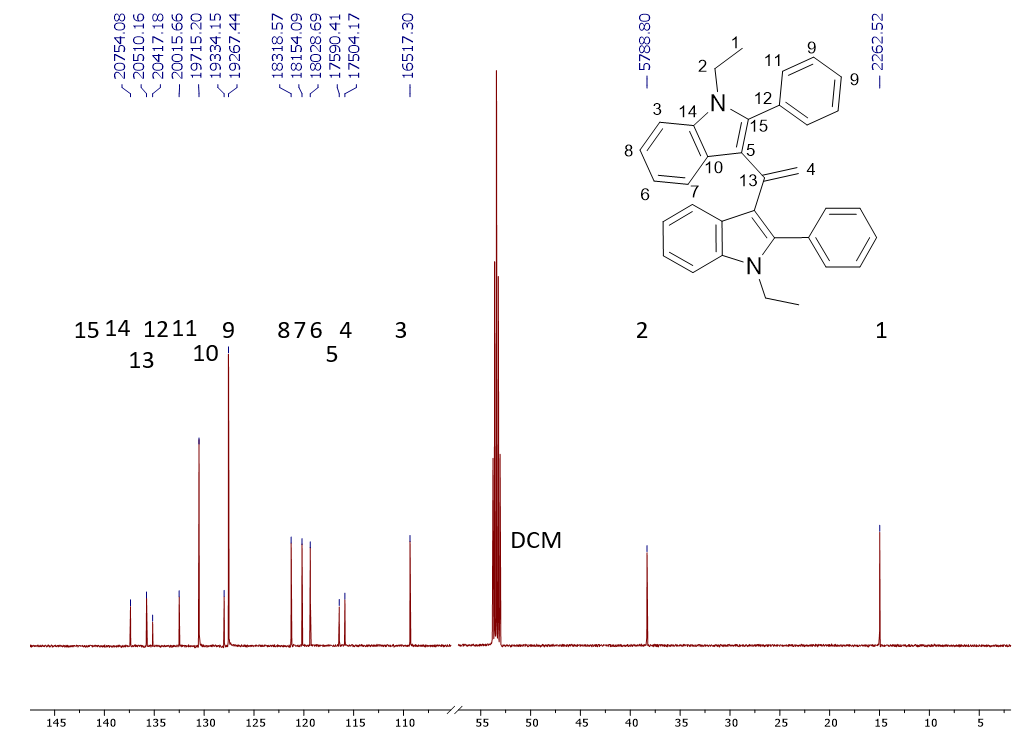
**

SI Fig. 12: ^13^C NMR characterisation of PID-1 in D_2_-DCM.

**
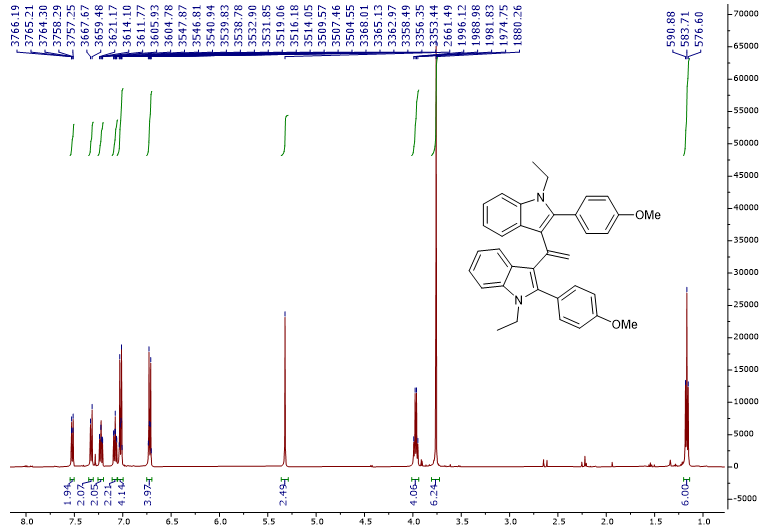
**

SI Fig. 13: ^1^H NMR spectrum of PID-2 in CDCl_3_.


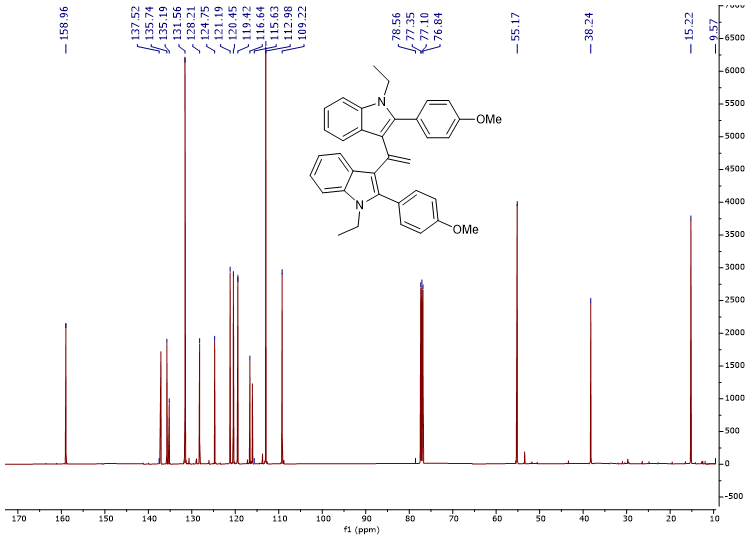


SI Fig. 14: ^13^C NMR spectrum of PID-2 in CDCl_3_.


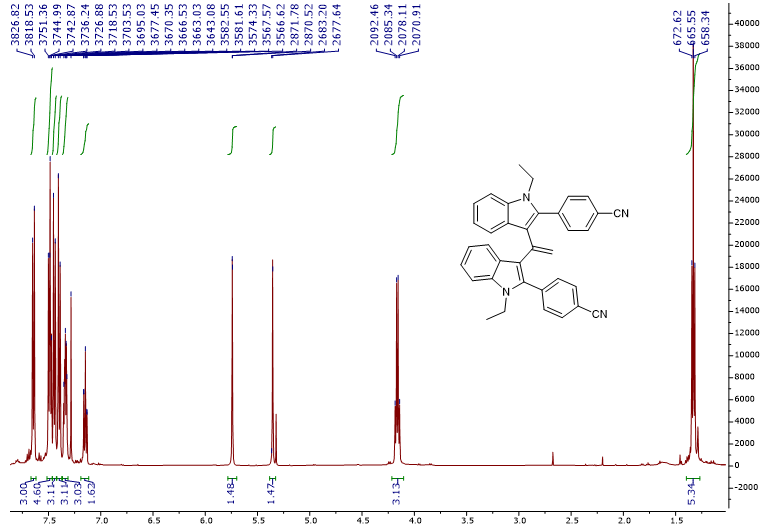


SI Fig. 15: ^1^H NMR spectrum of PID-3 in CDCl_3_


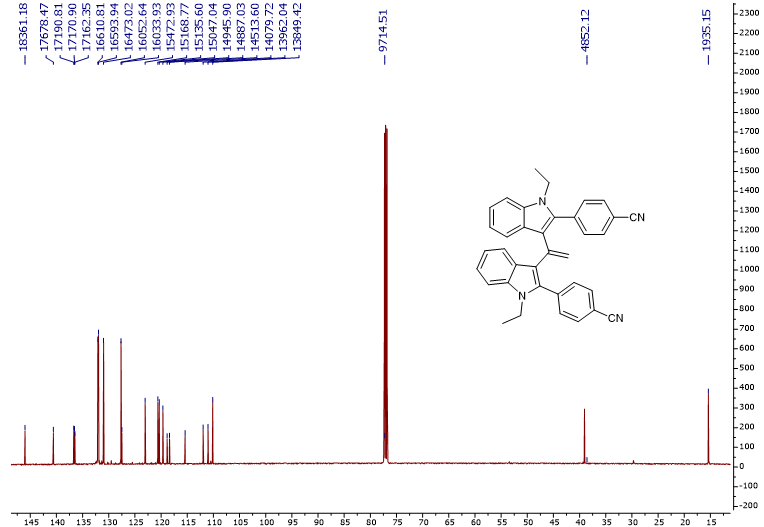


SI Fig. 16: ^13^C NMR spectrum of PID-3 in CDCl_3_.


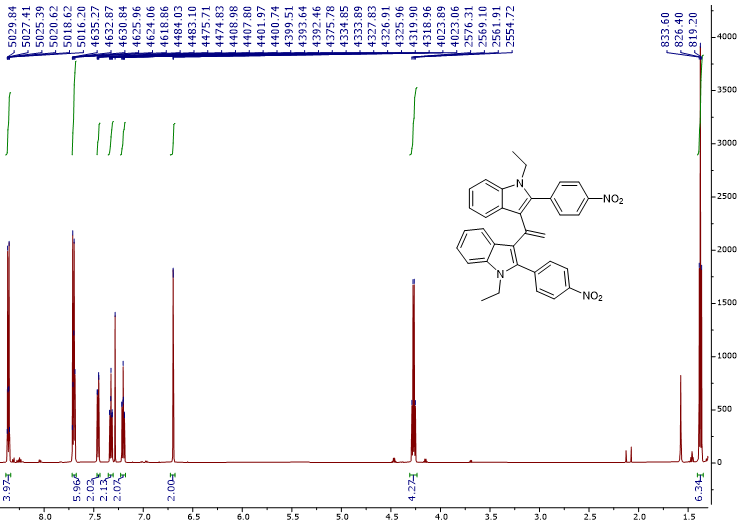


SI Fig. 17: ^1^H NMR spectrum of PID-4 in CDCl_3_.


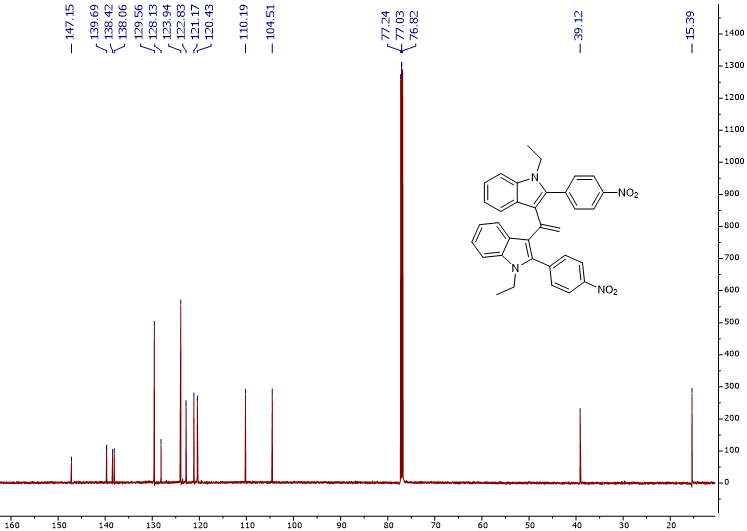


SI Fig. 18: ^13^C NMR spectrum of PID-4 in CDCl_3_.

**1.2.2 MS data for PID- 1, 2**

**
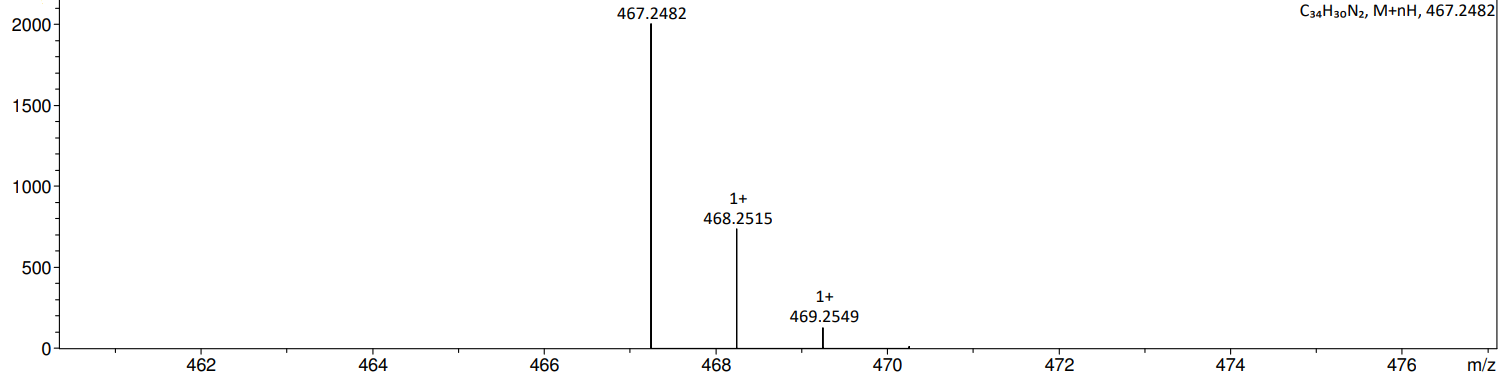
**

SI Fig. 19: HRMS of PID-1

**
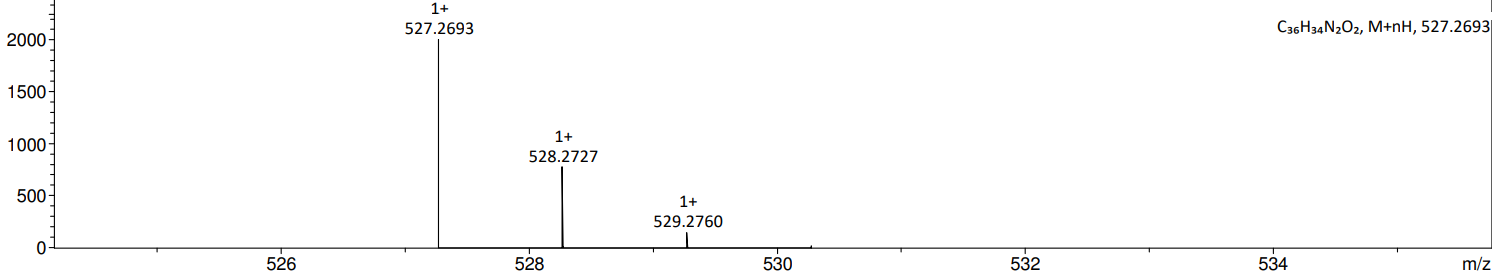
**

SI Fig. 20: HRMS of PID-2

**1.3 HPLC-ELSD analysis of compounds 1-4**

SI Fig. 21: HPLC-ELSD traces of PID-1-4.

**1.4 Thermal reversibility of PID-1 on paper**

**
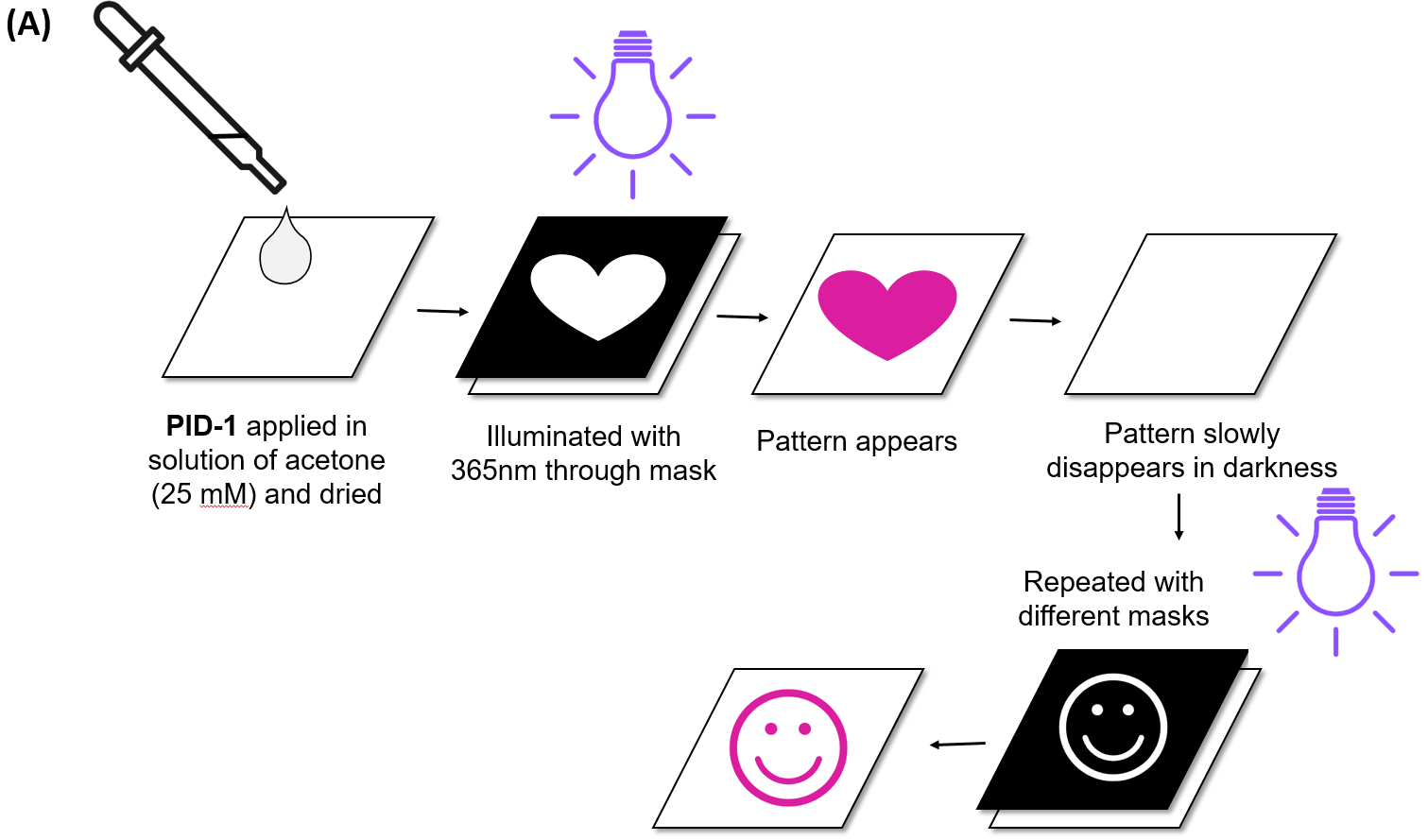
**

**
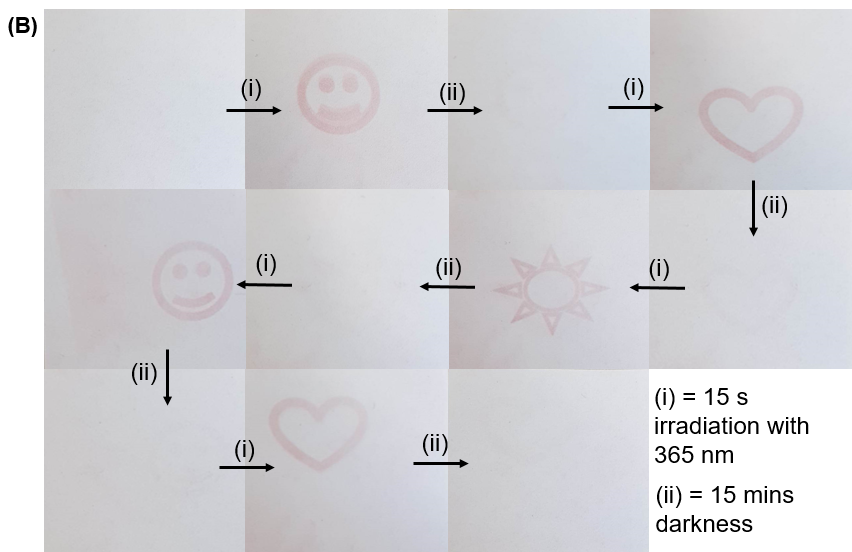
**

SI Fig. 22: (A) Patterning using 365 nm irradiation of PID-1 through a mask. (B) Cycles of the generation and disappearance of images upon irradiation. (i) 365 nm irradiation (15 s) and (ii) darkness (15 mins).

**1.5 Absorbance spectroscopy of thermal reversibility of thin film of PID-1**


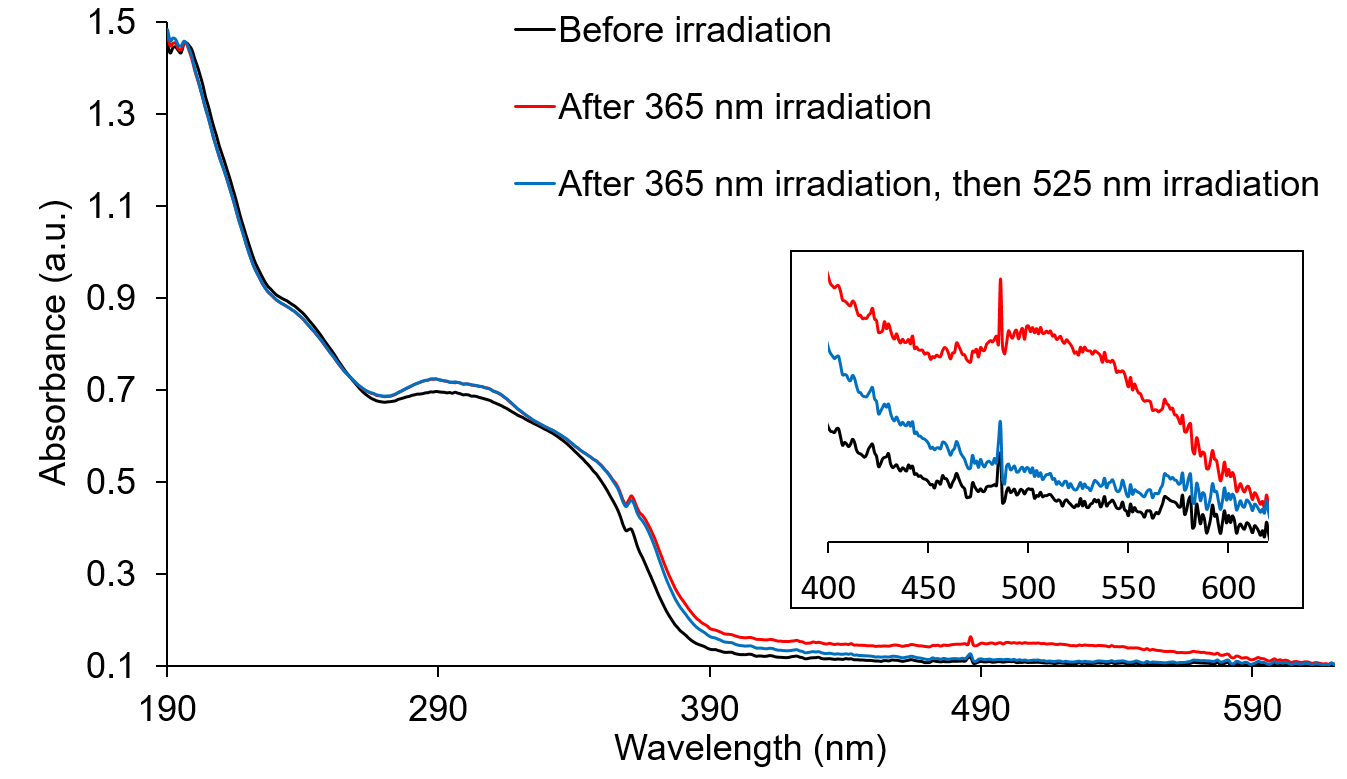


SI Fig. 23: Spectra of a thin film of PID-1 before irradiation, after 60 s irradiation at 365 nm, and after 60 s irradiation at 365 nm, then 15 s irradiation at 525 nm. Insert shows an expansion of the emerging band at 507 nm and its photo reversibility with irradiation at 525 nm

**
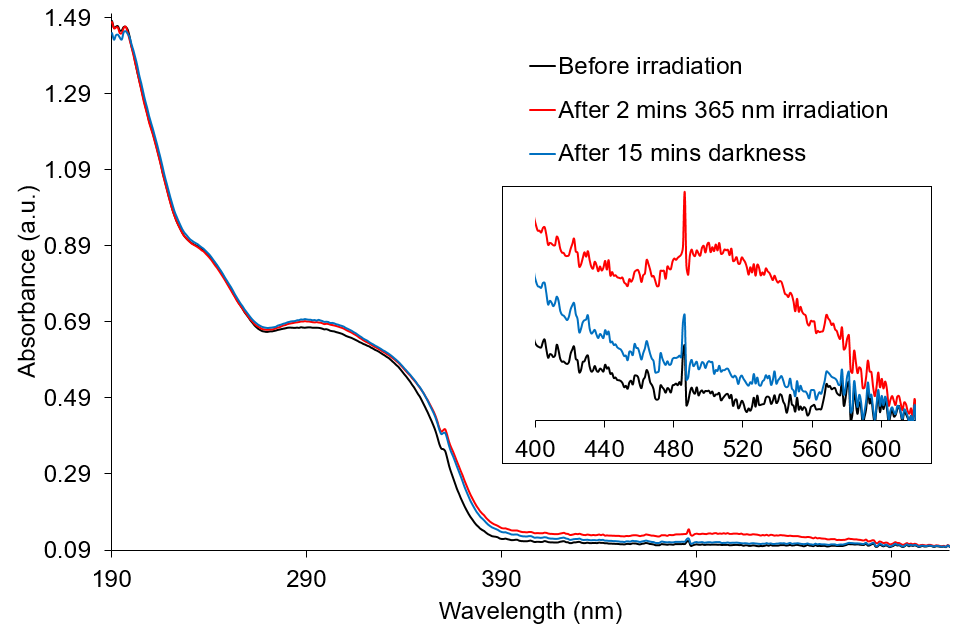
**

SI Fig. 24: Absorbance spectra of thin films of **PID-1**. Insert shows an expansion of the emerging band at 507 nm and its thermal reversibility.

**1.6 Absorbance spectroscopy of thin film of PID-3 with increasing irradiation**


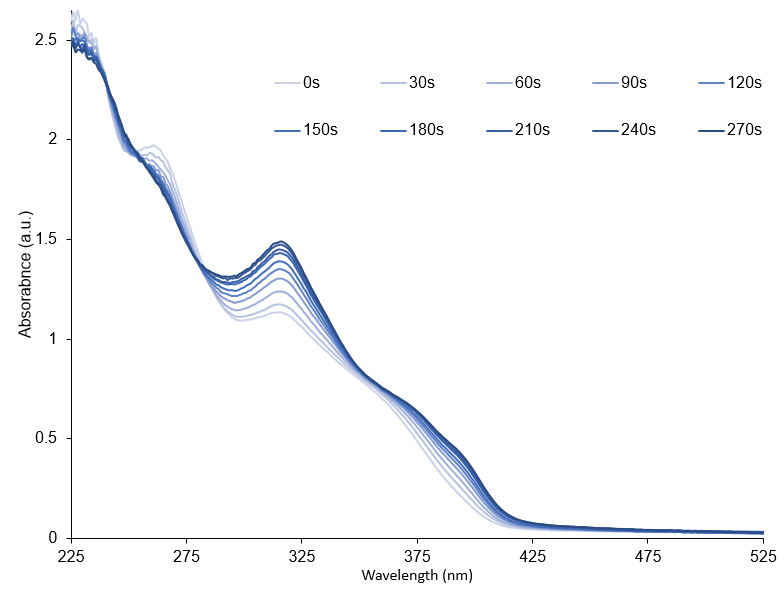


SI Fig 25: Absorbance spectrum of thin film of **PID-3** upon increasing irradiation at 365 nm.

**1.7 X-ray crystallography data**

**Reflection Statistics**

| Total reflections (after filtering) | 42587 | Unique reflections | 8583 |
| --- | --- | --- | --- |
| Completeness | 0.928 | Mean I/*σ* | 17.83 |
| hkl_max_ collected | (16, 18, 26) | hkl_min_ collected | (-15, -18, -28) |
| hkl_max_ used | (16, 18, 28) | hkl_min_ used | (-16, 0, 0) |
| Lim d_max_ collected | 100.0 | Lim d_min_ collected | 0.36 |
| d_max_ used | 5.97 | d_min_ used | 0.66 |
| Friedel pairs | 13938 | Friedel pairs merged | 1 |
| Inconsistent equivalents | 12 | R_int_ | 0.0436 |
| R_sigma_ | 0.0421 | Intensity transformed | 0 |
| Omitted reflections | 0 | Omitted by user (OMIT hkl) | 0 |
| Multiplicity | (19430, 9558, 1146, 107, 23, 10) | Maximum multiplicity | 15 |
| Removed systematic absences | 921 | Filtered off (Shel/OMIT) | 0 |

**Table 1**: Fractional Atomic Coordinates (×10^4^) and Equivalent Isotropic Displacement Parameters (Å^2^×10^3^) for **MB21001**. *U_eq_* is defined as 1/3 of the trace of the orthogonalised *U_ij_*.

| **Atom** | **x** | **y** | **z** | ***U_eq_*** |
| --- | --- | --- | --- | --- |
| N1 | 9624.5(8) | 3597.6(7) | 2284.1(5) | 15.44(18) |
| N2 | 5159.2(8) | 695.8(7) | 1357.8(5) | 15.02(18) |
| C1 | 9537.1(9) | 2981.7(8) | 1665.8(6) | 14.09(19) |
| C2 | 8777.6(9) | 2116.9(8) | 1750.3(6) | 13.33(19) |
| C3 | 8383.2(9) | 2196.5(9) | 2465.1(6) | 13.77(19) |
| C4 | 7684.7(10) | 1548.8(9) | 2889.7(6) | 16.7(2) |
| C5 | 7540.4(10) | 1852.9(10) | 3588.5(6) | 19.6(2) |
| C6 | 8061.9(11) | 2800.7(10) | 3875.4(6) | 20.9(2) |
| C7 | 8751.9(10) | 3454.7(10) | 3472.8(6) | 18.3(2) |
| C8 | 8920.2(9) | 3131.7(9) | 2774.6(6) | 14.9(2) |
| C9 | 10274.1(10) | 3263.8(9) | 1068.6(6) | 15.2(2) |
| C10 | 11555.2(10) | 3349.2(10) | 1194.4(7) | 20.6(2) |
| C11 | 12266.6(11) | 3592.7(11) | 636.7(7) | 24.6(3) |
| C12 | 11710.1(12) | 3747.5(11) | -49.3(7) | 26.4(3) |
| C13 | 10441.0(13) | 3669.2(11) | -179.0(7) | 26.9(3) |
| C14 | 9726.1(11) | 3432.9(10) | 379.2(6) | 20.6(2) |
| C15 | 10002.0(10) | 4716.0(9) | 2320.1(6) | 17.8(2) |
| C16 | 8937.1(12) | 5462.6(11) | 2123.4(8) | 27.3(3) |
| C17 | 6128.3(9) | 1378.6(8) | 1253.0(6) | 13.35(19) |
| C18 | 7206.0(9) | 804.0(8) | 1245.3(6) | 13.36(19) |
| C19 | 6891.7(10) | -296.6(8) | 1337.7(6) | 13.87(19) |
| C20 | 7562.3(10) | -1250.1(9) | 1393.3(6) | 18.0(2) |
| C21 | 6942.8(12) | -2194.5(9) | 1491.2(7) | 21.8(2) |
| C22 | 5662.8(12) | -2209.7(10) | 1526.6(7) | 22.9(2) |
| C23 | 4976.7(11) | -1284.1(9) | 1478.3(7) | 19.7(2) |
| C24 | 5607.2(10) | -331.7(8) | 1395.1(6) | 14.9(2) |
| C25 | 5952.0(9) | 2537.9(9) | 1173.1(6) | 14.7(2) |
| C26 | 5559.6(11) | 3156.1(10) | 1728.3(6) | 20.6(2) |
| C27 | 5451.9(12) | 4254.9(10) | 1652.1(7) | 24.7(3) |
| C28 | 5732.6(12) | 4741.8(10) | 1023.7(8) | 24.5(3) |
| C29 | 6113.0(12) | 4133.6(10) | 467.9(7) | 24.7(3) |
| C30 | 6216.6(11) | 3033.5(9) | 538.9(7) | 19.5(2) |
| C31 | 3852.1(10) | 959.7(10) | 1257.8(7) | 19.2(2) |
| C32 | 3331.8(13) | 829.2(13) | 486.2(8) | 31.3(3) |
| C33 | 8458.0(9) | 1251.2(8) | 1234.6(6) | 13.60(19) |
| C34 | 9253.2(11) | 842.7(9) | 799.7(7) | 18.8(2) |

**Table 2**: Anisotropic Displacement Parameters (×10^4^) for **MB21001**. The anisotropic displacement factor exponent takes the form: *-2π^2^[h^2^a*^2^ × U_11_+ ... +2hka* × b* × U_12_]*

| **Atom** | ***U_11_*** | ***U_22_*** | ***U_33_*** | ***U_23_*** | ***U_13_*** | ***U_12_*** |
| --- | --- | --- | --- | --- | --- | --- |
| N1 | 16.0(4) | 14.7(4) | 15.6(4) | -1.4(3) | 1.3(3) | -3.3(3) |
| N2 | 12.0(4) | 14.0(4) | 19.2(4) | -0.3(3) | 1.9(3) | -1.0(3) |
| C1 | 13.0(4) | 14.9(5) | 14.3(5) | -0.5(4) | 0.5(3) | -1.1(4) |
| C2 | 11.7(4) | 13.9(5) | 14.3(5) | 0.2(4) | 0.9(3) | 0.1(3) |
| C3 | 12.4(4) | 14.5(5) | 14.4(5) | 1.1(4) | 1.1(3) | 1.5(4) |
| C4 | 14.3(4) | 16.7(5) | 19.2(5) | 3.6(4) | 2.6(4) | 1.0(4) |
| C5 | 17.8(5) | 23.3(6) | 18.4(5) | 6.2(4) | 4.8(4) | 3.3(4) |
| C6 | 20.2(5) | 27.9(6) | 14.6(5) | 0.3(4) | 2.6(4) | 5.5(4) |
| C7 | 17.3(5) | 21.3(5) | 16.0(5) | -2.5(4) | -0.7(4) | 2.7(4) |
| C8 | 13.1(4) | 16.7(5) | 14.8(5) | 1.4(4) | 0.5(3) | 1.1(4) |
| C9 | 16.2(4) | 12.9(5) | 16.7(5) | 0.0(4) | 3.2(4) | -2.5(4) |
| C10 | 16.5(5) | 22.6(6) | 22.8(6) | 3.2(4) | 2.5(4) | -1.0(4) |
| C11 | 17.1(5) | 28.2(6) | 29.3(6) | 1.2(5) | 6.8(4) | -3.1(4) |
| C12 | 26.6(6) | 29.4(7) | 24.5(6) | -0.6(5) | 10.4(5) | -8.3(5) |
| C13 | 29.4(6) | 34.3(7) | 17.2(5) | 1.9(5) | 3.2(5) | -10.8(5) |
| C14 | 18.6(5) | 24.5(6) | 18.5(5) | 1.0(4) | 1.1(4) | -7.5(4) |
| C15 | 17.7(5) | 14.7(5) | 20.4(5) | -2.0(4) | -1.3(4) | -3.0(4) |
| C16 | 23.3(6) | 20.3(6) | 37.0(7) | 4.8(5) | -4.3(5) | 1.1(5) |
| C17 | 13.7(4) | 13.1(5) | 13.3(4) | -0.4(3) | 1.4(3) | -0.4(3) |
| C18 | 13.8(4) | 12.1(4) | 14.2(5) | -0.8(4) | 1.4(3) | -0.8(3) |
| C19 | 15.2(4) | 12.5(5) | 13.9(4) | -1.0(4) | 1.2(3) | -1.7(4) |
| C20 | 17.6(5) | 14.9(5) | 21.3(5) | -1.9(4) | 0.2(4) | 0.7(4) |
| C21 | 26.2(6) | 13.2(5) | 26.1(6) | -1.0(4) | 2.6(4) | 1.2(4) |
| C22 | 28.0(6) | 14.5(5) | 26.8(6) | -1.4(4) | 5.7(5) | -5.6(4) |
| C23 | 19.1(5) | 17.6(5) | 22.8(5) | -2.1(4) | 4.6(4) | -5.2(4) |
| C24 | 16.2(4) | 13.2(5) | 15.3(5) | -1.2(4) | 1.7(4) | -1.1(4) |
| C25 | 13.5(4) | 13.7(5) | 16.7(5) | 0.3(4) | -0.1(4) | 0.5(4) |
| C26 | 26.8(6) | 18.6(5) | 16.5(5) | -1.2(4) | 2.1(4) | 2.8(4) |
| C27 | 30.1(6) | 18.0(6) | 25.6(6) | -5.0(5) | 0.1(5) | 4.3(5) |
| C28 | 25.5(6) | 14.2(5) | 33.3(7) | 0.7(5) | -0.6(5) | 3.2(4) |
| C29 | 27.4(6) | 18.2(6) | 29.2(6) | 6.9(5) | 5.6(5) | 1.6(5) |
| C30 | 21.4(5) | 17.6(5) | 20.1(5) | 1.7(4) | 5.6(4) | 2.8(4) |
| C31 | 12.8(4) | 21.1(6) | 23.9(6) | -1.7(4) | 2.4(4) | 0.8(4) |
| C32 | 24.0(6) | 37.4(8) | 30.6(7) | -4.4(6) | -9.2(5) | 2.6(6) |
| C33 | 13.6(4) | 11.9(4) | 15.3(5) | 1.0(4) | 1.3(3) | -1.2(3) |
| C34 | 18.6(5) | 16.6(5) | 22.0(5) | -1.8(4) | 5.9(4) | -1.4(4) |

**Table 3**: Bond Lengths in Å for **MB21001**.

| **Atom** | **Atom** | **Length/Å** | |
| --- | --- | --- | --- |
| N1 | C1 | 1.3890(14) |  |
| N1 | C8 | 1.3780(14) |  |
| N1 | C15 | 1.4639(14) |  |
| N2 | C17 | 1.3883(13) |  |
| N2 | C24 | 1.3798(14) |  |
| N2 | C31 | 1.4598(14) |  |
| C1 | C2 | 1.3835(14) |  |
| C1 | C9 | 1.4762(15) |  |
| C2 | C3 | 1.4448(15) |  |
| C2 | C33 | 1.4759(15) |  |
| C3 | C4 | 1.4071(15) |  |
| C3 | C8 | 1.4142(15) |  |
| C4 | C5 | 1.3855(17) |  |
| C5 | C6 | 1.4060(18) |  |
| C6 | C7 | 1.3814(17) |  |
| C7 | C8 | 1.3965(16) |  |
| C9 | C10 | 1.4007(15) |  |
| C9 | C14 | 1.3911(16) |  |
| C10 | C11 | 1.3882(17) |  |
| C11 | C12 | 1.3862(19) |  |
| C12 | C13 | 1.3878(18) |  |
| C13 | C14 | 1.3895(17) |  |
| C15 | C16 | 1.5133(17) |  |
| C17 | C18 | 1.3805(14) |  |
| C17 | C25 | 1.4749(15) |  |
| C18 | C19 | 1.4384(15) |  |
| C18 | C33 | 1.4783(14) |  |
| C19 | C20 | 1.4026(15) |  |
| C19 | C24 | 1.4150(15) |  |
| C20 | C21 | 1.3854(16) |  |
| C21 | C22 | 1.4034(17) |  |
| C22 | C23 | 1.3814(17) |  |
| C23 | C24 | 1.3953(15) |  |
| C25 | C26 | 1.3945(16) |  |
| C25 | C30 | 1.3934(16) |  |
| C26 | C27 | 1.3915(17) |  |
| C27 | C28 | 1.3841(19) |  |
| C28 | C29 | 1.3836(19) |  |
| C29 | C30 | 1.3919(17) |  |
| C31 | C32 | 1.5149(18) |  |
| C33 | C34 | 1.3422(15) |  |

**Table 4**: Bond Angles in ^°^ for **MB21001**.

| **Atom** | **Atom** | **Atom** | **Angle/^°^** | |
| --- | --- | --- | --- | --- |
| C1 | N1 | C15 | 124.86(9) |  |
| C8 | N1 | C1 | 108.50(9) |  |
| C8 | N1 | C15 | 123.22(9) |  |
| C17 | N2 | C31 | 126.05(9) |  |
| C24 | N2 | C17 | 108.33(9) |  |
| C24 | N2 | C31 | 123.82(9) |  |
| N1 | C1 | C9 | 119.71(9) |  |
| C2 | C1 | N1 | 109.90(9) |  |
| C2 | C1 | C9 | 130.24(10) |  |
| C1 | C2 | C3 | 106.32(9) |  |
| C1 | C2 | C33 | 127.84(10) |  |
| C3 | C2 | C33 | 125.80(9) |  |
| C4 | C3 | C2 | 134.67(10) |  |
| C4 | C3 | C8 | 118.30(10) |  |
| C8 | C3 | C2 | 106.93(9) |  |
| C5 | C4 | C3 | 119.07(11) |  |
| C4 | C5 | C6 | 121.27(11) |  |
| C7 | C6 | C5 | 121.10(11) |  |
| C6 | C7 | C8 | 117.42(11) |  |
| N1 | C8 | C3 | 108.34(9) |  |
| N1 | C8 | C7 | 128.83(10) |  |
| C7 | C8 | C3 | 122.80(10) |  |
| C10 | C9 | C1 | 119.45(10) |  |
| C14 | C9 | C1 | 121.44(9) |  |
| C14 | C9 | C10 | 119.10(10) |  |
| C11 | C10 | C9 | 120.32(11) |  |
| C12 | C11 | C10 | 119.99(11) |  |
| C11 | C12 | C13 | 120.14(12) |  |
| C12 | C13 | C14 | 119.98(12) |  |
| C13 | C14 | C9 | 120.46(11) |  |
| N1 | C15 | C16 | 112.13(10) |  |
| N2 | C17 | C25 | 122.02(9) |  |
| C18 | C17 | N2 | 109.74(9) |  |
| C18 | C17 | C25 | 128.23(10) |  |
| C17 | C18 | C19 | 106.81(9) |  |
| C17 | C18 | C33 | 126.14(10) |  |
| C19 | C18 | C33 | 126.65(9) |  |
| C20 | C19 | C18 | 134.55(10) |  |
| C20 | C19 | C24 | 118.75(10) |  |
| C24 | C19 | C18 | 106.68(9) |  |
| C21 | C20 | C19 | 118.95(10) |  |
| C20 | C21 | C22 | 121.09(11) |  |
| C23 | C22 | C21 | 121.39(11) |  |
| C22 | C23 | C24 | 117.36(11) |  |
| N2 | C24 | C19 | 108.39(9) |  |
| N2 | C24 | C23 | 129.19(10) |  |
| C23 | C24 | C19 | 122.42(10) |  |
| C26 | C25 | C17 | 121.33(10) |  |
| C30 | C25 | C17 | 119.53(10) |  |
| C30 | C25 | C26 | 119.12(11) |  |
| C27 | C26 | C25 | 120.30(11) |  |
| C28 | C27 | C26 | 120.18(12) |  |
| C29 | C28 | C27 | 119.91(12) |  |
| C28 | C29 | C30 | 120.23(12) |  |
| C29 | C30 | C25 | 120.26(11) |  |
| N2 | C31 | C32 | 112.25(10) |  |
| C2 | C33 | C18 | 115.92(9) |  |
| C34 | C33 | C2 | 123.40(10) |  |
| C34 | C33 | C18 | 120.61(10) |  |

**Table 5**: Torsion Angles in ^°^ for **MB21001**.

| **Atom** | **Atom** | **Atom** | **Atom** | **Angle/^°^** |  |
| --- | --- | --- | --- | --- | --- |
| N1 | C1 | C2 | C3 | -0.65(12) | |
| N1 | C1 | C2 | C33 | -178.39(10) | |
| N1 | C1 | C9 | C10 | 56.10(15) | |
| N1 | C1 | C9 | C14 | -124.81(12) | |
| N2 | C17 | C18 | C19 | 1.02(12) | |
| N2 | C17 | C18 | C33 | -172.13(10) | |
| N2 | C17 | C25 | C26 | 63.44(14) | |
| N2 | C17 | C25 | C30 | -118.49(12) | |
| C1 | N1 | C8 | C3 | 0.53(12) | |
| C1 | N1 | C8 | C7 | 178.47(11) | |
| C1 | N1 | C15 | C16 | 84.81(14) | |
| C1 | C2 | C3 | C4 | -175.10(11) | |
| C1 | C2 | C3 | C8 | 0.95(11) | |
| C1 | C2 | C33 | C18 | -146.86(11) | |
| C1 | C2 | C33 | C34 | 36.25(17) | |
| C1 | C9 | C10 | C11 | 178.70(11) | |
| C1 | C9 | C14 | C13 | -178.23(12) | |
| C2 | C1 | C9 | C10 | -118.94(13) | |
| C2 | C1 | C9 | C14 | 60.14(17) | |
| C2 | C3 | C4 | C5 | 176.25(11) | |
| C2 | C3 | C8 | N1 | -0.91(11) | |
| C2 | C3 | C8 | C7 | -179.01(10) | |
| C3 | C2 | C33 | C18 | 35.81(15) | |
| C3 | C2 | C33 | C34 | -141.08(12) | |
| C3 | C4 | C5 | C6 | 0.96(16) | |
| C4 | C3 | C8 | N1 | 175.90(9) | |
| C4 | C3 | C8 | C7 | -2.20(16) | |
| C4 | C5 | C6 | C7 | -0.93(17) | |
| C5 | C6 | C7 | C8 | -0.64(16) | |
| C6 | C7 | C8 | N1 | -175.45(11) | |
| C6 | C7 | C8 | C3 | 2.23(16) | |
| C8 | N1 | C1 | C2 | 0.09(12) | |
| C8 | N1 | C1 | C9 | -175.89(9) | |
| C8 | N1 | C15 | C16 | -71.85(14) | |
| C8 | C3 | C4 | C5 | 0.55(15) | |
| C9 | C1 | C2 | C3 | 174.78(10) | |
| C9 | C1 | C2 | C33 | -2.96(18) | |
| C9 | C10 | C11 | C12 | -0.31(19) | |
| C10 | C9 | C14 | C13 | 0.85(18) | |
| C10 | C11 | C12 | C13 | 0.6(2) | |
| C11 | C12 | C13 | C14 | -0.1(2) | |
| C12 | C13 | C14 | C9 | -0.6(2) | |
| C14 | C9 | C10 | C11 | -0.40(18) | |
| C15 | N1 | C1 | C2 | -159.46(10) | |
| C15 | N1 | C1 | C9 | 24.56(15) | |
| C15 | N1 | C8 | C3 | 160.49(9) | |
| C15 | N1 | C8 | C7 | -21.57(17) | |
| C17 | N2 | C24 | C19 | 2.03(12) | |
| C17 | N2 | C24 | C23 | -178.31(11) | |
| C17 | N2 | C31 | C32 | 85.08(14) | |
| C17 | C18 | C19 | C20 | -178.10(12) | |
| C17 | C18 | C19 | C24 | 0.22(12) | |
| C17 | C18 | C33 | C2 | 46.83(15) | |
| C17 | C18 | C33 | C34 | -136.19(12) | |
| C17 | C25 | C26 | C27 | 177.16(11) | |
| C17 | C25 | C30 | C29 | -176.85(11) | |
| C18 | C17 | C25 | C26 | -115.97(13) | |
| C18 | C17 | C25 | C30 | 62.09(15) | |
| C18 | C19 | C20 | C21 | 179.16(12) | |
| C18 | C19 | C24 | N2 | -1.39(12) | |
| C18 | C19 | C24 | C23 | 178.93(10) | |
| C19 | C18 | C33 | C2 | -124.99(11) | |
| C19 | C18 | C33 | C34 | 51.99(16) | |
| C19 | C20 | C21 | C22 | 0.76(18) | |
| C20 | C19 | C24 | N2 | 177.25(10) | |
| C20 | C19 | C24 | C23 | -2.44(17) | |
| C20 | C21 | C22 | C23 | -1.2(2) | |
| C21 | C22 | C23 | C24 | -0.19(19) | |
| C22 | C23 | C24 | N2 | -177.61(11) | |
| C22 | C23 | C24 | C19 | 2.01(17) | |
| C24 | N2 | C17 | C18 | -1.92(12) | |
| C24 | N2 | C17 | C25 | 178.57(10) | |
| C24 | N2 | C31 | C32 | -77.84(15) | |
| C24 | C19 | C20 | C21 | 0.99(17) | |
| C25 | C17 | C18 | C19 | -179.51(10) | |
| C25 | C17 | C18 | C33 | 7.34(18) | |
| C25 | C26 | C27 | C28 | 0.07(19) | |
| C26 | C25 | C30 | C29 | 1.26(17) | |
| C26 | C27 | C28 | C29 | 0.5(2) | |
| C27 | C28 | C29 | C30 | -0.1(2) | |
| C28 | C29 | C30 | C25 | -0.75(19) | |
| C30 | C25 | C26 | C27 | -0.92(17) | |
| C31 | N2 | C17 | C18 | -167.02(10) | |
| C31 | N2 | C17 | C25 | 13.47(16) | |
| C31 | N2 | C24 | C19 | 167.54(10) | |
| C31 | N2 | C24 | C23 | -12.80(18) | |
| C33 | C2 | C3 | C4 | 2.70(19) | |
| C33 | C2 | C3 | C8 | 178.75(10) | |
| C33 | C18 | C19 | C20 | -5.0(2) | |
| C33 | C18 | C19 | C24 | 173.33(10) | |

**Table 6**: Hydrogen Fractional Atomic Coordinates (×10^4^) and Equivalent Isotropic Displacement Parameters (Å^2^×10^3^) for **MB21001**. *U_eq_* is defined as 1/3 of the trace of the orthogonalised *U_ij_*.

| **Atom** | **x** | **y** | **z** | ***U_eq_*** |
| --- | --- | --- | --- | --- |
| H26 | 5388(15) | 2814(13) | 2179(9) | 30(4) |
| H4 | 7312(14) | 900(12) | 2689(8) | 21(4) |
| H10 | 11942(15) | 3237(13) | 1689(9) | 29(4) |
| H15A | 10356(14) | 4870(12) | 2812(9) | 24(4) |
| H14 | 8834(16) | 3395(13) | 304(9) | 32(4) |
| H30 | 6490(15) | 2621(13) | 149(9) | 30(4) |
| H7 | 9105(15) | 4108(13) | 3675(9) | 25(4) |
| H34A | 8984(14) | 279(12) | 449(9) | 23(4) |
| H20 | 8443(14) | -1254(11) | 1361(8) | 18(4) |
| H15B | 10653(14) | 4809(13) | 1998(9) | 25(4) |
| H31A | 3738(14) | 1694(12) | 1434(8) | 21(4) |
| H32A | 3781(19) | 1311(16) | 158(11) | 48(6) |
| H31B | 3428(15) | 487(13) | 1576(9) | 27(4) |
| H34B | 10096(15) | 1091(13) | 799(9) | 26(4) |
| H23 | 4097(15) | -1311(13) | 1507(9) | 28(4) |
| H12 | 12199(16) | 3908(14) | -455(10) | 35(4) |
| H11 | 13156(15) | 3665(13) | 729(9) | 31(4) |
| H13 | 10035(16) | 3790(14) | -667(10) | 39(5) |
| H27 | 5155(16) | 4672(14) | 2044(10) | 37(5) |
| H29 | 6308(16) | 4482(14) | 14(10) | 36(5) |
| H21 | 7420(16) | -2880(14) | 1535(9) | 33(4) |
| H6 | 7959(15) | 3003(13) | 4362(9) | 30(4) |
| H16A | 9237(16) | 6218(14) | 2138(10) | 35(5) |
| H16B | 8298(18) | 5405(15) | 2461(11) | 42(5) |
| H22 | 5257(15) | -2920(14) | 1603(9) | 31(4) |
| H32B | 2430(20) | 1012(16) | 445(11) | 51(6) |
| H32C | 3434(17) | 95(17) | 337(11) | 46(5) |
| H5 | 7059(15) | 1395(13) | 3875(9) | 26(4) |
| H16C | 8568(18) | 5298(15) | 1646(11) | 42(5) |
| H28 | 5686(16) | 5512(14) | 966(9) | 34(4) |
